# Advances in Functionalized Photosensitive Polymeric Nanocarriers

**DOI:** 10.3390/polym13152464

**Published:** 2021-07-27

**Authors:** Maritza Fernández, Jahir Orozco

**Affiliations:** Max Planck Tandem Group in Nanobioengineering, University of Antioquia, Complejo Ruta N, Calle 67 N° 52-20, Medellín 050010, Colombia; maritza.fernandez@udea.edu.co

**Keywords:** functional nanoparticle, photoresponsive nanoparticle, light-responsive drug delivery, polymer nanocarrier

## Abstract

The synthesis of light-responsive nanocarriers (LRNs) with a variety of surface functional groups and/or ligands has been intensively explored for space-temporal controlled cargo release. LRNs have been designed on demand for photodynamic-, photothermal-, chemo-, and radiotherapy, protected delivery of bioactive molecules, such as smart drug delivery systems and for theranostic duties. LRNs trigger the release of cargo by a light stimulus. The idea of modifying LRNs with different moieties and ligands search for site-specific cargo delivery imparting stealth effects and/or eliciting specific cellular interactions to improve the nanosystems’ safety and efficacy. This work reviews photoresponsive polymeric nanocarriers and photo-stimulation mechanisms, surface chemistry to link ligands and characterization of the resultant nanosystems. It summarizes the interesting biomedical applications of functionalized photo-controlled nanocarriers, highlighting the current challenges and opportunities of such high-performance photo-triggered delivery systems.

## 1. Introduction

Particular interest has emerged in designing and synthesizing smart nanocarriers that respond to specific stimuli. Among them, LRNs offer outstanding opportunities for controlled drug delivery in the frontier of physic, chemistry, biology, and converging engineering fields [1,2]. Such smart nanocarriers can be functionalized with different moieties and/or ligands at the outermost surface (or inner structure) to impart stealth effect and/or elicit specific cellular interactions to improve the safety and efficacy of the resultant nanosystems [3,4].

Drug delivery systems triggered by external stimuli allow precise control over the timing, dosage, and drug release location from the physician or the patient. Many external stimuli such as light, ultrasound, electricity, and magnetic signals have been investigated [2]. Nevertheless, light is a particularly appealing choice due to its easiness of control and manipulation and the long successful history of applying light to trigger a therapy. Along with high biocompatibility, the possibility of spatiotemporal control of therapeutic agents and convenience, LRNs enjoy other remarkable features like their release profiles can be regulated by adjusting light wavelength, power intensity, duration of exposure, and beam diameter [5]. A broad range of stimuli-responsive nanocarriers with diverse sizes, shapes, and surface properties have been designed in this context [1,5]. They include liposomes, polymer nanoparticles, micelles, dendrimers, and inorganic nanoparticles made of iron oxide, quantum dots, gold, or metal oxide frameworks [6]. The carriers’ size is typically small, from a few tenths to a few hundred nanometers and the shape, composition, and surface chemistry can be tailored on-demand to achieve the desired LRNs [7].

Polymer nanoparticles can be loaded with active compounds entrapped within the core or adsorbed/linked onto the polymeric surface [8]. The advantages of polymer nanoparticles as active principle delivery systems over other particular systems include high drug encapsulation efficiency, intracellular uptake, stability of encapsulated active substances, biocompatibility and biodegradability with tissue and cells, especially when prepared from biopolymers [9,10]. The polymeric nanocarriers can be nanocapsules, composed of an oily core in which the drug is usually dissolved, dispersed, or embedded, surrounded by a polymeric shell that controls the release profile; and nanospheres, based on a continuous polymeric network in which the drug can be retained inside or adsorbed onto their surface [11,12].

The light source for LRNs can vary from ultraviolet (UV) (200–400 nm), visible (Vis) (400–700 nm), and near-infrared (NIR) (700–1000 nm) [6]. The controlled drug release can be achieved through photo-isomerization, e.g., azobenzene and spiropyran (SP), photo-cleavage, e.g., coumarin-based groups, photo-crosslinking, and photoinduced rearrangement mechanisms, e.g., coumarin and cinnamoyl [13]. These features, along with other materials’ physical and chemical properties, ranging from wettability, degradability, and electrostatics to permeability and mechanical resistance, have made these LRNs have a wide range of practical applications [14,15]. 

Despite the multiple applications of LRNs at the biomedical level, the modification of surfaces with specific targeting ligands is often necessary to reduce toxicity and increase stability and bioavailability [3]. Functionalization of nanoparticles (NPs) is the process of changing their surface chemistry by grafting chemical functional groups or molecules to achieve new capabilities, characteristics, properties, or functions [4,16]. Chemical modification of the LRNs surface is a step-by-step process that requires physicochemical and functional characterization for each step. NPs have mainly been functionalized with thiols, disulfides, amines, nitriles, carboxylic acids, carbonyls, sulfhydryls, azides, hydroxyls, phosphines, and biomolecules [17]. Surface modification can be achieved by non-covalent strategies based on weak hydrogen bond, electrostatic, ionic, van der Walls and hydrophobic interactions, absorption, entrapment, and layer-by-layer approaches, etc. [18,19,20]. Non-covalent interactions have the advantage of being relatively simple and do not affect either the molecules’ structure or their interaction with biological targets but can be easily influenced by different variables, such as pH and ionic strength [21]. In contrast, covalent interactions directly bind the molecule of interest to the NP’s reactive moieties. This approach involves a linkage reaction aided by a catalyst and is the choice over physical adsorption when long-term stability is required [16].

**Figure 1:**
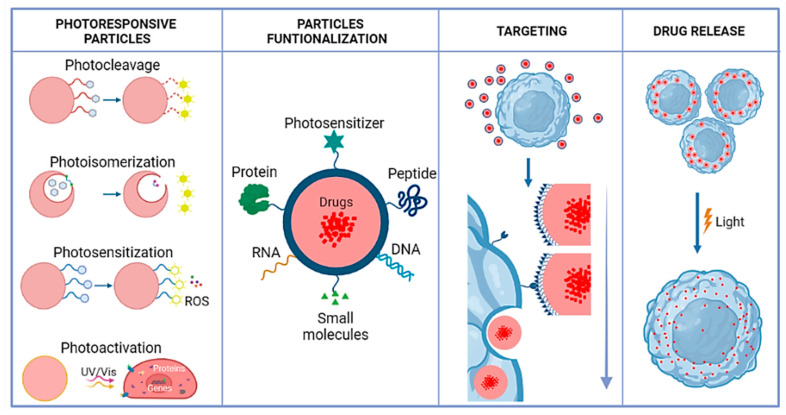
Schematic illustration of photosensitive polymeric nanocarriers: photosensitive mechanism, functional nanocarriers, nanocarriers targeting, and drug release.

This review provides an overview of current advances in LRN drug delivery systems (Figure 1). It summarizes different light-responsive mechanisms, including photocleavage, photoisomerization, and photo cross-linking/-decross-linking. It discusses NIR for nanoscale drug delivery systems, focusing on the chemical aspects and practical examples of the functionalization process. The challenges and future perspectives of developing LRNs in controlled drug delivery applications are also assessed.

## 2. Photoresponsive Polymeric Nanocarriers

### 2.1. Photo-Stimulation Mechanisms (Cleavage, Isomerization, Cross/Decross-Linking)

**Figure 2:**
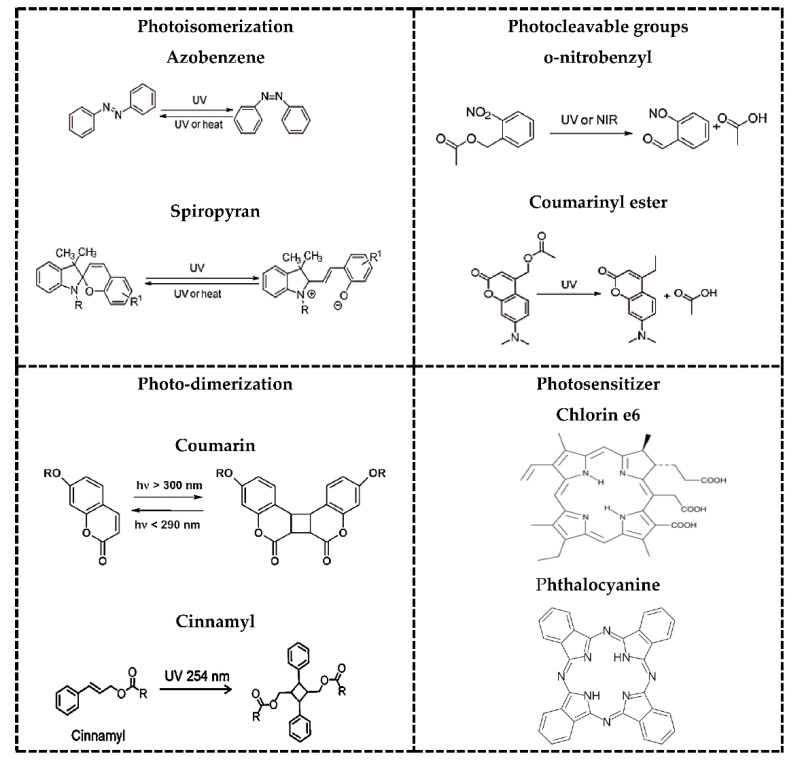
Photoresponsive molecules for phototriggered drug release.

Owing to their non-invasiveness and the possibility of remote spatiotemporal control, a large variety of photoresponsive systems have been engineered in the past few years to achieve on-demand drug release in response to illumination at a specific wavelength in the UV, visible, or NIR regions of the electromagnetic spectrum [22,23]. Photoactivatable protecting groups include photoremovable, photoreleasable, or photocleavable [24], which must exhibit some characteristics. Sufficient absorption of the irradiated light that must either not be absorbed by other molecules or not trigger unwanted photochemical transformations in the system of interest. It should release protected species within a time frame compatible with the application and be soluble and stable in the targeted medium/environment (an aqueous solution in typical biological/medical applications). It should not produce reactive or toxic side-products upon irradiation and be detectable in the medium, for example, by light emission [25]. Additionally, photoresponse mechanisms are typically photoinduced and depend upon the type of photosensitive moiety and the constituents’ polymer interaction nature, thereby responding to light reversibly or irreversibly. Photoresponsive mechanisms can be generally classified in photocleavage, photoisomerization, and photocross-linking/-decross-linking [23]. In Figure 2, there are some examples of photoresponsive molecules for phototriggered targeting.

### 2.2. Photocleavage

Polymers that respond to light irreversibly are primarily via photocleavage of photolabile groups incorporated in their structure or through irreversible photo-cross-linking. The o-nitrobenzyl (ONB) group is one of the most useful photolabile moieties for photoresponsive polymeric systems used for biomedical purposes [1,2,3]. The mechanism of photolysis of ONB derivatives has been suggested to involve a rearrangement reaction to form an acinitro intermediate, which cleaves the carbon-heteroatom bond at the benzylic position to generate a nitrosophenyl carbonyl compound (aldehyde or ketone) and a leaving group such as an alcohol, amine, or carboxylic acid [26]. Four types of photocleavage can be distinguished. 

Self-immolate polymers (SIPs) with photocleavable terminal groups, produced through a polymerization reaction of the appropriate monomer to cap the polymer’s terminal head-group with a specific protecting group leading to a polymer with a trigger. The protecting group’s selective cleavage would then initiate the sequential polymer fragmentation into the original building blocks from head to tail. Therefore, the appropriate selection of the trigger would allow the design of many different responsive materials [27]. 

Light-degradable polymers with photocleavable side chains. Photocleavable species are conjugated to the polymer side chains as protecting groups of nucleophiles, such as alcohols, thiols, and amines. Upon light irradiation, the light-responsive units’ photocleavage leads to the deprotection of these functional groups, which can initiate a consecutive degradation of polymer main chains and release the polymer nanoparticles’ payload [26]. 

Light-induced hydrophobic to hydrophilic transition of polymers with photocleavable side chains, after removing photoresponsive protecting groups, the light-triggered polymers’ hydrophobicity changes undergo hydrophobic-to-hydrophilic transitions [6,14]. These hydrophobicity changes lead to nanoparticle degradation, followed by the payload drug release [5].

SIPs with multiphotocleavable linkers. By incorporating multiple photocleavable units into polymer main chains, the polymer chain responds to UV-light irradiation and then degrades at each junction point. The more photocleavable groups a polymer chain contains, the more thoroughly the polymer decomposes [27].

### 2.3. Photoisomerization

The most widely cited examples of phototriggered isomerization are based on azobenzenes, which switch from the thermodynamically favored trans-conformation to the bent cis-conformation upon irradiation with UV light. Azobenzene contains an azo and an aryl group, with cis and trans isomers. It shows a significant π-π transition in the UV region and a faint π-π transition in the visible region. Therefore, upon irradiation to UV light, the trans structure of azobenzene changes into the cis structure. Additionally, azobenzene can reverse from the cis to the trans structure [28]. Azobenzenes are the most widely used groups because they are of easy synthesis, relatively high photostationary states and quantum yields, fast photoisomerization, and a low rate of photobleaching [29]. SP is another well-known photochromic switch with a hydrophobic cis moiety able to isomerize to hydrophilic trans conformation, merocyanine (MC), under UV irradiation via intramolecular carbon-oxygen bond breakage. SP can show a light-driven change in fluorescence properties from ring open to closed-form reversibly. Ring closed form is colorless SP and ring open form is colored MC. The SP and MC forms of different physical and chemical properties [22] are sensitive to, e.g., UV light (365 nm) and visible light (550 nm), respectively. 

### 2.4. Photocross-Linking-Decross-Linking

Light-induced reversible dimerization describes the process in which two previously unbound molecules are covalently coupled to each other reversibly. Some compounds have been reported to have such reversible dimerization properties, e.g., cinnamylidene acetate [30], nitrocinnamate [31], and anthracene [32]. However, cinnamic acid and coumarin derivatives are the most frequently employed molecules that can undergo reversible photodimerization. The decross-linking process occurs spontaneously when the nanocarriers are irradiated with a shorter wavelength light (higher energy photons). Despite many reports on light-sensitive nanosystems, the superficial penetration depth (<200 mm) caused by the strong scattering of soft tissues has restricted the UV-based systems for in vivo applications. Moreover, even in in vitro applications, the UV light causes genetic damage to the cells [22].

### 2.5. NIR Light 

An alternative to circumvent UV light stimulated systems limitations might be the NIR irradiation (700–900 nm), which can penetrate more deeply into soft tissues without causing damage in the area of application [23,33,34]. Currently, the capability of plasmonic NPs to transduce the absorbed NIR light into heat has been adopted to trigger the release of drug molecules. Moreover, it was reported that a photothermal effect of Au nanorods upon NIR irradiation could cause a fast increase of local temperature, which can be exploited to induce denaturation of DNA helices attached to the nanocarriers and thereby release drug molecules bound to consecutive cytosine-guanine base pairs [1,35]. 

## 3. Common Ligands Involved in Active Targeting 

The cellular transport/uptake of NPs is highly dependent on their intrinsic properties such as size, shape, morphology, and extrinsic ones from surface modifications [20]. For example, the hydrophobic character of Nps can reduce the circulation time in vivo because of opsonization, i.e., small molecules called opsonins adsorbed onto the surface of NPs favors phagocytosis [17]. Besides, the surface charge could impact their uptake; i.e., positively charged NPs induce higher complement activation than negatively and neutrally charged NPs [10,15]. Therefore, it is desirable to synthesize NPs directly from block polymers having both hydrophobic and hydrophilic fragments and neutral pending moieties to increase hydrophobic drug loading while prolonging the particles’ circulation time [9,36,37].

The modulation of cell-extracellular matrix binding controls the cell life in complex tissues. Cells respond to micro-and nano-features with different chemistries and topographies, changing cell alignment, polarization, elongation, migration, proliferation, and gene expression [38]. The cells express many molecules in their membrane, fundamental for an active targeting with NPs conjugated to a targeting moiety that can bind on purpose to the surface of specific cell types [39]. Therefore, developing synthetic materials and technological strategies that enable adequate cell stimulus mimicking is a promising alternative for drug delivery. They include physical artificial scaffolds to mimic naturally appearing complex structures or micro and nanostructures to stress the cell cytoskeleton or modulate substrate stiffness; and proteins and growth factors [40]. 

The choice of a targeting ligand revolves around numerous considerations, including availability, easiness of production, diversity, affinity, protocols for conjugation, immunogenicity, and cost. These parameters should be carefully considered when designing NPs with a maximum targeting capacity while minimizing the cost. Independently of conjugation approaches and target cell characteristics, NPs can be functionalized by targeting ligands of different nature, such as aptamers, DNA or RNA, small molecules, peptides, antibodies, or glycoproteins.

### 3.1. Nucleic Acids 

#### 3.1.1. DNA

DNA-functionalized NPs (DNA-NPs) were first reported in 1996 by Mirkin et al., who described a synthetic strategy that enabled the preparation of nucleic acid-NPs consisting of densely functionalized and highly oriented DNA strands covalently attached to the core surface of gold NPs (AuNPs) [41]. DNA is the most robust and amenable for chemical modifications of the nucleotide sequences, sugars, or phosphate backbones, thus providing a vast diversity of options of chemical functionality [42]. 

Functional DNA molecules include aptamers, DNAzymes, and aptazymes obtained by in vitro selection with the Systematic Evolution of Ligands by Exponential Enrichment (SELEX) technology. Moreover, DNA exhibits many favorable features for biological applications such as small size, low immunogenicity, easiness of synthesis and chemical modification, and facile surface functionalization [43]. Integration of DNA into NPs imparts specific recognition capabilities to NPs for various targets, ranging from small molecules to biomacromolecules and even viruses or cells [42]. Importantly, DNA-based probes are highly biocompatible and non-toxic to cells. Various methods have been used to functionalize the DNA onto NPs surfaces, such as covalent conjugation, dative bonding, and electrostatic interaction [44,45]. 

#### 3.1.2. RNA

The messenger RNA (mRNA), transfer RNA (tRNA), and ribosomal RNA (rRNA) have been studied in the frame of the drug-release concept [43]. However, RNA is highly vulnerable to serum exo- and endo-nucleases, leading to a short half-life in serum. In the last year, some of them have been used for biomedical applications, e.g., RNA interference (RNAi), which functions in a cellular process whereby short RNAs mediate the silencing of a particular gene [46]. Silencing is mediated by small interfering RNAs (siRNA) in which the antisense strand of a double-stranded RNA duplex guides recognition and catalytic degradation of a target mRNA by the RNA-induced silencing complex (RISC). Silencing is also mediated by endogenous short RNAs ~20–25 nt known as microRNAs (miRNAs) that either repress translation and/or enhance target mRNAs’ degradation. There has been tremendous interest in advancing the fundamental understanding of both pathways and harnessing them for therapeutic applications by delivering short RNAs into cells to control gene expression [43,47,48]. For example, siRNA has been chemically conjugated to various bioactive molecules, lipids, polymers, peptides, and inorganic nanostructured materials to enhance their pharmacokinetic behavior, cellular uptake, target specificity, and safety [5]. 

#### 3.1.3. Aptamers

Single-stranded oligonucleotides with sizes between 70 and 100 nucleotides are generated by SELEX and form unique tertiary structures that allow specific interactions with target molecules [43,44,49]. Aptamer truncation has the potential to reduce the cost of drug manufacturing as they can be obtained through chemical synthesis, avoiding using animals or cells, with high reproducibility and capacity to be marked, facilitating material quality assurance, and preventing unexpected toxicity. Aptamers prefer to interact with positively charged surfaces of the target proteins due to the negatively charged nature of backbone linkages, they should be highly stable in the body and the nucleotide length can be sufficiently shortened. Other characteristics include stability at room temperature and amenability to modify its structure (pegylation, etc.) [46,49,50].

### 3.2. Peptides and Proteins

The limited ability of current drugs to penetrate cells has contributed to their lack of biological action, poor therapeutic efficacy and high toxicity. Such limitations have stimulated developing alternative strategies to site-specific direct NPs, e.g., through proteins or peptides. Various small proteins or peptides have been conjugated on the NPs surface to improve drugs’ selectivity [38,51]. Unlike proteins, peptides with a linear or cyclic sequence of amino acids (not more than 50 residues) target NPs moieties by folding into three-dimensional structures conferring high stability and resistance in the cellular environment [16,52]. They also enjoy other desirable features, including low molecular weight, tissue penetration capabilities, easiness of production and relative flexibility in chemical conjugation processes. The family arginine–glycine–aspartic acid (RGD), such as cyclic RGD (cRGD), are widely used for conjugation with NPs [44,53,54]. Some protein transduction domains of peptides, such as cell-penetrating peptides (CPPs), generally with a maximum length of 30 amino acid residues, e.g., TAT or polyArg can translocate cell membranes efficiently without compromising their integrity [55]. These CPPs are characterized by a high content of basic amino acid residues, resulting in an overall positive net charge. CPPs are advantageous over other translocation methods because they possess high cellular permeability rates, the ability to translocate into a broad spectrum of cell types, large cargo capacity and low cell toxicity associated with no immunological response [56].

#### Antibodies

They are very large glycoproteins (150 kDa) whose principal role is to recognize antigens from the circulation, facilitating the activity of the macrophages, conferring high immunogenicity to the NPs after conjugation [21,57]. Monoclonal antibodies (MAbs) are macromolecules widely used to target ligands because of their immediate availability and high affinity and specificity for molecular targets [21,53,58]. In addition, these ligands usually exhibit high binding affinity. However, the bulky size and constant redundant region may cause the generation of immunogenicity as size increases. The use of antibody fragments, nanobodies, affibodies, and peptides may help overcome this shortcoming [20].

### 3.3. Small Molecules

Small molecules are identified as organic molecules with less than 500 Da molecular weight. With other properties, such as high stability, chemical management, and low-cost production, their small size confers them optimum characteristics for specific targeting when conjugated with NPs [1,59,60]. The most extended examples are folic acid (FA) [37,61,62], small molecules, or polymers like dextran, starch, citrate, or poly(ethylene glycol) (PEG). NPs, when coated with such small molecules, give rise to nanoprobes with enhanced biodistribution, longer circulation time, and efficient cellular uptake [63]. For their use as potential delivery systems in vivo, the aforementioned NPs must have long plasma half-lives. In this sense, PEG is the most widely used macromolecule to prolong nanocarriers’ half-life. PEG strongly affects nanoparticle structure, stabilization, and biodistribution in vitro and in vivo [64,65]. These long-circulating NPs can circulate for a prolonged period and target a particular organ carrying DNA in gene therapy or delivering proteins, peptides, and drugs [66,67,68]. For systemic applications, developing surface-functionalized and long-circulating NPs as cellular probes and delivery agents is highly desired for passive targeting tumors and inflammatory sites [69]. The simple structure and chemical stability of PEG make it an inert and biocompatible polymer and PEG-modified NPs affords long-circulation by evading macrophage-mediated uptake and removal from the systemic circulation [64,65].

## 4. Effect of the Protein Corona

NPs are immediately covered with proteins generating the so-called protein corona [70]. The physicochemical properties of the NPs highly influence the composition of the protein corona formed on the NPs surface. Consequently, the corona is considered one of the significant players affecting the biological interactions of the NPs, including cytotoxicity, cell uptake, and transport. Additionally, the correlation between NPs properties and their cellular transport/uptake appears to be cell type-dependent, indicating that different kinds of mechanisms could take place [63,71,72]. 

This process is reversible and in some cases, the proteins can be rearranged by forcing them into a specific orientation over the NP surface [72]. The preservation of ligand shell structure and composition is essential to conserve the selectivity and sensitivity, while nonspecific protein adsorption must be minimized. Some authors had shown that the surface charge of NPs influences their interactions with biological systems. Generally, neutral and negatively charged NPs show lower interaction with plasma proteins than positively charged ones that interact strongly with blood components, undergo nonspecific binding and cause cell lysis [63,70]. 

PEG linking is currently the most popular approach to rendering the NP surface inert toward nonspecific adsorption. Its properties as hydrophilicity, conformational flexibility, charge neutrality, and steric repulsion are responsible for its bio-invisible (stealth) nature [73]. As a proof-of-concept, Li et al., (2018) explored a photoinduced PEG deshielding nanocarrier TK-NPCe6&PTX to circumvent the challenge above. The TK-NPCe6&PTX encapsulating chlorin e6 (Ce6) and paclitaxel (PTX) were self-assembled from an innovative thioketal (TK) linkage-bridged diblock copolymer of PEG with poly (d,l-lactic acid) (PEG-TK-PLA). They demonstrated that the high PEGylation of TK-NPCe6&PTX in the blood helps the nanocarrier efficiently avoids rapid clearance and consequently prolongs its circulation time. At the desired site (tumor), 660-nm red light irradiation led to ROS generation in situ [74]. Gangopadhyay et al. [75] developed a new organic polymer based on 4-arm PEG for more specific treatment towards tumor tissues, where biotin was covalently attached to the 4-arm PEG targeting pendant. The NPs contained coumarin fluorophore, which rendered synergistic treatment to Hela cell line via the concomitant occurrence of photodynamic therapy (PDT) and chemotherapy. PEG-Bio-Cou-Cbl NPs released almost 80% of anticancer drug chlorambucil upon exposure to UV/vis light of ≥365 nm. In vitro application of PEG-Bio-CouCbl NPs in cancerous Hela cell line suggested more significant cell damage in the combined effect of PDT and chemotherapy than only PDT, while biotin conjugation ensured better accumulation of the NPs in Hela cell lines than in non-cancerous L929 cells.

## 5. Functionalization of Photosensitive Nanocarriers

Ligands may be reversibly adsorbed or entrapped to and retained or embedded into LRNs through ionic, electrostatic, hydrogen bonding, hydrophobic, or van der Waals interactions [76] or irreversibly through covalent linking. Interactions depend not only on LRNs composition and reactive functional groups but also on the ligand’s chemical nature, affinity, isoelectric point (pI), and the polarity of solvent and media conditions. The type and composition of the cross-linkers and surface chemistry are also crucial to consider when functionalizing LRNs [4]. Their selection depends on the NPs features, the reaction (interaction) efficiency expected and other specific conditions needed to occur, as functionalizing NPs by different paths may lead to different outcomes. For example, for antibody NPs coating, physical adsorption may show low reproducibility and poor stability at different pH conditions. In contrast, linking ligands to NPs by covalent bonding through various linking chemistries offers high stability despite possible aggregation, polymerization, and random antibody orientation [77]. The approach holds the potential to increase the intracellular cargo concentration, decrease dose and cargo side effects, thereby improving a therapeutic regime’s effectiveness. Itraconazole (ITZ) was encapsulated into functional polymeric NPs based on poly(lactic acid-co-glycolic acid) (PLGA) polymers for their targeted and controlled release into macrophages. Although the nanocarriers were not photosensitive, the work exemplifies how they were functionalized with the F4/80 antibody and compares the functionalization extent by the adsorption and EDC/NHS methods. The approach demonstrated to increase macrophage uptake in vitro and the nanosystem’s efficacy to eliminate the *Histoplasma capsulatum* fungus, paving the way towards developing highly efficient nanocarriers for drug delivery against intracellular infections [78].

### 5.1. Nonspecific Adsorption

Nonspecific adsorption is the simplest and modification-free method for immobilizing biomolecules at NP surfaces, mainly based on physical adsorption or ionic binding [20]. In the first case, the biomolecules are attached to the NPs through weak interactions, while in the second one, they are bound through stronger ionic linkages. However, such non-covalent immobilization processes could be reversed by changing the pH, ionic strength, temperature, polarity of the solvent, or interaction with other molecules in biological samples. Another drawback is the need for a high concentration of biomolecules, which could be expensive (e.g., antibodies). Besides, denaturation may occur in the linking process, changing the biomolecule native 3D structure, with the concomitant irreversible loss of the biological activity [16,56]. Yet, grafting multi-charged polymers on the NP surface facilitates ionic interaction with biomolecules and enlarges the coating extent by promoting a 3D multipoint ionic adsorption within the grafted polymer matrix. Therefore, although these methods are straightforward and cost affordable, they must be selected only whenever biomolecule leakage from NPs is not crucial. Otherwise, covalent immobilization is a better option [77,79].

The pH, temperature, incubation time, and antibody: NPs ratio are parameters that influence the extent of NP functionalization by physical adsorption. For example, we recently reported how the pH, away of the biomolecule pI, increased the functionalized NPs size and polydispersity index (PDI) and decreased the ζ-potential, suggesting agglomeration, precipitation of non-absorbed and possibly denatured antibodies but destabilizing NPs [77]. However, at the optimal conditions of antibody: NPs ratio, pH and incubation temperature and time, the maximum surface coverage was high enough with relatively higher NPs stability than the other evaluated pHs [9,21,76]. 

### 5.2. Entrapment

Along with adsorption and covalent bonding, drugs and ligands can also be entrapped into 3D cross-linked networks from natural or synthetic polymeric matrices such as nanogels. Such drug delivery systems enjoy swellability, appropriate porosity, tunable size, and large surface area [30]. They are amenable for multiple bio-conjugations for encapsulation of mostly hydrophilic drugs with low cytotoxicity, high loading capacity, physiological stability and stimuli-triggered drug release. Again, electrostatic interactions, hydrogen bonding, or van der Waals interactions govern drug molecule-nanogel interactions. Hydrogels allow that the 3D-conformational structure of biomolecules used as ligands or drugs remains almost unaltered after the entrapment process impacting the resultant nanobioconjugates efficiency and efficacy. Nanogels based on chitosan assembled with light-responsive polymers are the most extended in this category [31,80,81]. 

A nanogel-based system based on chitosan and poly(N-isopropylacrylamide) (PNIPAM) was synthesized by radical polymerization and modified with Au and magnetic NPs to reach a light-responsive drug delivery nanocarrier [82]. AuNPs were synthesized in situ on chitosan surfaces, serving as photothermal transducers, leading to spherical and uniform nanogels with high photothermal conversion ability. The surface plasmon resonance of AuNPs from green light irradiation generated a local heat to the nanogels shrink due to the PNIPAM molecules and drug molecules released. The resultant intelligent visible light-sensitive drug nanogel holds the potential to reduce the side effects and toxicity of free drugs in the body. 

### 5.3. Polymer Coating

#### 5.3.1. Covalent Immobilization of Drugs and Ligands

It is a chemical process of joining two or more molecules at the NPs structure by covalent interactions. It uses cross-linking reagents that contain distal reactive groups to link pending reactive moieties of NPs, drugs and ligands [3,56]. Similar chemistry is applied to amino acids and nucleic acids surface modification and labeling. 

#### 5.3.2. Cross-Linkers

**Figure 3:**
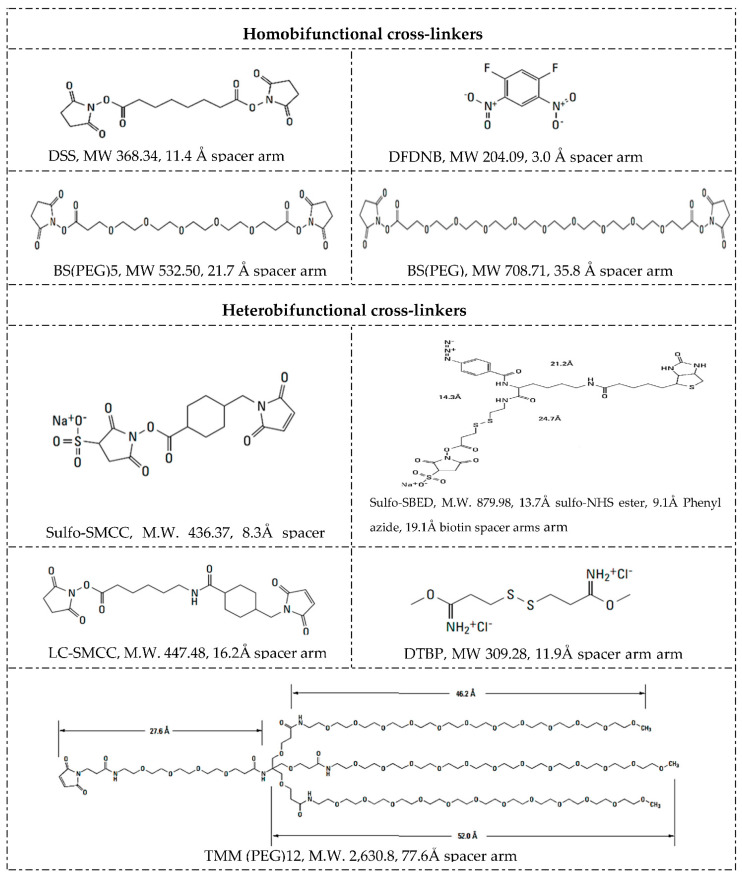
Structures and characteristics of some cross-linkers.

Along with NPs, drugs and linkers, the cross-linker defines the method and mechanism for chemical functionalization. Cross-linkers contain at least two reactive chemical groups that interact with the NPs on one terminus and the drug or ligands on the other end, acting as a bridge in between. Selection of the proper cross-linker depends on their properties and reaction conditions such as pH, buffer, and NP, drug or ligand concentrations [58]. For example, the composition of the spacer, water solubility, and cell membrane permeability. Spacer arm length and functional groups in branched or straight chains and cleavage sites between the reactive groups. Whether the cross-linker is homobifunctional, heterobifuncional, or trifunctional, with the same or different reactive groups at either end, Figure 3 summarizes different cross-linkers commonly used in the covalent immobilization of ligands at LRNs [56,77,83]. 

The chemical chain between two reactive groups is called the spacer arm, whose length can go from zero-length to >100 angstroms, determining how flexible the linking will be. Longer spacer arms are more flexible and reduce steric hindrance but have more potential sites for nonspecific binding. For example, length spacer arms of 1,5-difluoro-2,4-dinitrobenzene (DFDNB), disuccinimidyl suberate (DSS), bis(succinimidyl) penta(ethylene glycol) (BS(PEG)_5_), and bis(succinimidyl) nona(ethylene glycol) (BS(PEG)_9_) are 3.0, 11.4, 21.7, and 35.8 Å, respectively [56,84,85,86]. 

Homobifunctional cross-linkers have the same reactive groups at either end of a spacer arm and are generally used in one-step reactions to functionalize NPs with biomolecules randomly. Heterobifunctional cross-linkers have different reactive groups at either end and can be used either in a single-step reaction or sequential two-step conjugations that minimize undesirable polymerization and self-conjugation. DSS is an extended simple cross-linker with identical amine-reactive NHS ester groups at either end of a short spacer arm. In contrast, sulfosuccinimidyl 4-(*N*-maleimidomethyl)cyclohexane-1-carboxylate (Sulfo-SMCC) is a standard linker, having an amine-reactive sulfo-NHS ester group at one end and a sulfhydryl reactive maleimide group distally within a cyclohexane spacer arm, amenable for sequential two-step conjugation reactions [37,56]. 

Trifunctional cross-linkers typically possess two chemically reactive functional groups and one label such as a biotin group, e.g., sulfo-*N*-hydroxysuccinimidyl-2-(6-[biotinamido]-2-(p-azido benzamido)-hexanoamido) ethyl-1,3′-dithioproprionate (sulfo-SBED). As a way to illustrate the importance of arm’s length, biofunctionalized mesoporous silica NPs loaded with cargo were assessed as a drug delivery strategy to multidrug-resistant cells [87]. NPs were functionalized by covalent attachment of streptavidin onto their surface via a heterobifunctional cross-linker with an extended 5 kDa PEG spacer arm (NHS-PEG-maleimide) or with shorter arm cross-linkers such as succinimidyl-4-(*N*-maleimidomethyl)cyclohexane-1-carboxy-(6-amidocaproate) (LC-SMCC). It is important to note that bioconjugation with a long arm (5 kDa) PEG cross-linker maximized the targetability of the NPs. 

Cross-linkers’ spacer arm may be designed to have cleavage sites built into to break stable, covalent NP-cross-linker-ligand bonds and recover the individual component counterparts. The disulfide bridge is a commonly used cleavage site that can be readily reduced with reducing agents such as ß-mercaptoethanol, dithiothreitol (DTT) or tris(2-carboxyethyl)phosphine (TCEP) [88,89]. Dimethyl 3,3′ dithiobispropionimidate · 2HCl (DTBP) is a typical example of a cleavage cross-linker. The molecular composition affects solubility and nonspecific binding [90,91]. Not water-soluble spacer arms of cross-linkers contain hydrocarbon chains and those water-soluble ones commonly have PEG chains. Cross-linkers containing hydrophobic uncharged hydrocarbon chains typically require an organic solvent such as dimethyl sulfoxide (DMSO) or *N*,*N*-dimethylformamide (DMF) to dissolve, better suited for penetrating the cell membrane and performing intercellular cross-linking. Adding a charged sulfonate group to their termini forms a soluble analog, e.g., whereas DSS is soluble in organic solvents, its BS_3_ derivate is water-soluble. BS(PEG)_5_ is also soluble in aqueous buffers because of its PEG spacer. PEGylations also allow either straight, e.g., BS(PEG)_5_ and BS(PEG)_9_, or branched—(methyl-PEG_12_)_3_-PEG_4_-maleimide (TMM(PEG)_12_)—modifications [92,93]. Figure 3 shows the structures of some cross-linkers.

Cross-linking is mostly at near-physiologic conditions to maintain the native structure of the biomolecule complex, whose optimal cross-linker-to-ligand molar ratio must be determined empirically [79,83,94]. It is critical to consider the degree of conjugation depending on the application. For example, when linking an antibody or an enzyme to NPs, a low-to-moderate degree of conjugation is desired to retain their biological activity. The number of available surface functional groups is also worthy of consideration. A lower cross-linker-to-protein ratio is enough for a high number of target groups; for a limited number of potential target groups, a higher cross-linker-to-protein ratio may be required [56,86]. Excess of reagents may be avoided to elude unnecessary cross-linking processes and interfering subproducts. As an illustrative example, it is worth mentioning glutaraldehyde (GA), one of the most widely used homobifunctional cross-linkers. Hybrid nanospheres based on chitosan/gold nanorods (CS-AuNR) were successfully synthesized, the cisplatin anticancer drug loaded into the spherical matrix and GA introduced to cross-link the hybrid nanospheres [95]. They were utilized as contrast agents for real-time cell imaging while serving as a NIR thermotherapy approach for irradiation-induced cancer cell death and effectively attacking the cancer cells. The work demonstrated the feasibility of using such an all-in-one system for simultaneous cancer cell imaging and attacking and inducing cancer cell death. Table 1 summarizes manners to functionalize photosensitive nanocarriers, pointing out advantages, disadvantages and main interactions.

### 5.4. Functional and Reactive Groups 

Functionalization of NPs is the process of changing their surface chemistry by grafting chemical functional groups or molecules on their surface to achieve new capabilities, characteristics, properties, or functions. Covalent coupling of ligands to NP surfaces involves a chemical reaction between reactive functional groups from the nanostructured platform and free-functional groups from the ligand. Reactive functional groups include carboxylic acid (-COOH), aldehyde (-CHO), amine (-NH_2_), sulfhydryl (-SH), photo-reactive hydroxyl (-OH), and azide (-N_3_), which target common free-functional groups from ligands such as cysteine and lysine residues from proteins and amino and carboxylic moieties from synthetically modified nucleic acids and peptides [4,17,56]. 

#### 5.4.1. Carboxylic Acid (R-COOH)

Carboxylic acid is present on the protein structure’s surface at the C-terminus of each polypeptide chain and side chains of aspartic and glutamic acids, highly reactive towards carbodiimides, one of the most common coupling reaction. Among carbodiimides, *N*-(3-Dimethylaminopropyl)-*N*′-ethylcarbodiimide hydrochloride (EDC) is a zero-length cross-linker that reacts with carboxylic acid groups to form an active unstable O-acylisourea intermediate, naturally displaced by a nucleophile from primary amino groups forming an amide link with the original carboxyl group and releasing the EDC as a soluble urea by-product (see reaction 1 in Table 2) [56]. Direct EDC-mediated cross-linking usually causes random polymerization of polypeptides due to the multiple carboxyls and amines of peptides and proteins. N-hydroxysuccinimide (NHS) and its water-soluble analog (sulfo-NHS) are often introduced in EDC coupling reactions to improve efficiency or create dry-stable (amine-reactive) intermediates [96,97] (see reaction 2 in Table 2). EDC couples NHS to carboxyl groups, forming a reactive NHS-ester, more stable than the O-acylisourea intermediate, with efficient conjugation to primary amines at physiological pH. EDC may also activate 5′ phosphate groups in the presence of imidazole for conjugation to primary amines. Unlike EDC, which cross-links carboxylic acids to primary amines in direct biomolecules conjugation processes, not aqueous-soluble dicyclohexyl carbodiimide (DCC) does it when conjugated to NPs but before linking to the ligand [96]. Because the reaction is water-free, the resulting NHS-ester can be prepared and stabilized as a dried powder without appreciable hydrolysis, being very useful in commercial peptide synthesis [98] (see reaction 3 in Table 2).

**Figure 4:**
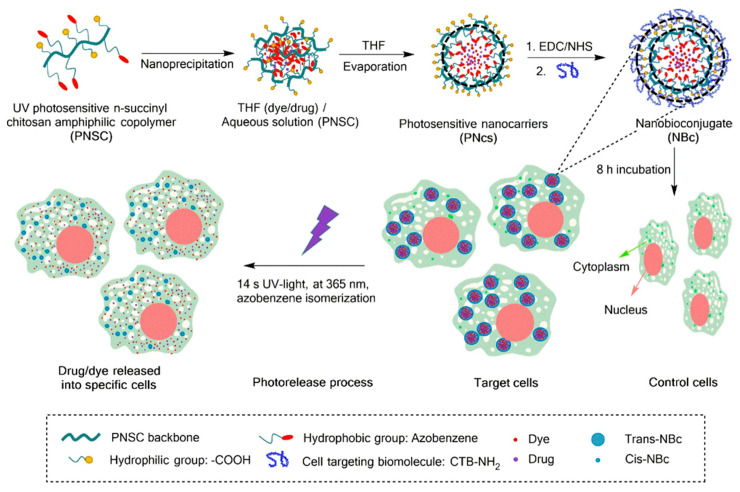
Illustration of PNc synthesis, nanocarrier self-assembly, cargo co-encapsulation, and further functionalization with the CTB. Selective uptake of the resultant nanobioconjugate and UV-light induced cargo release into cardiomyocytes by an isomerization process of the azobenzene photosensitive molecule-containing pothosensitive nanocarrier. Reprinted with permission from [99]. Copyright 2020 Springer Nature.

As an illustration of the EDC/NHS coupling chemistry, our group got encapsulated dofetilide into azobenzene-modified chitosan polymer NPs for their specific photo-delivery into cardiomyocyte target cells. Primary amines of a fluorescein isothiocyanate (FITC) targeting transmembrane peptide (CTB) were linked to carboxylic acid moieties, exposed at the outermost nanocarrier’s surface, by the EDC/NHS covalent coupling in two steps. The improved cellular uptake was explained by the peptide-coated nanocarriers’ capacity to disrupt the cardiomyocyte’s cell membrane. Therefore, intracellular cargo release was dramatically accelerated upon short UV-light irradiation (Figure 4) [99].

#### 5.4.2. Carbonyl (R-CHO and R-C-O-R’) 

The polarity of carbonyls, especially in aldehydes, makes the carbon atom electrophilic and reactive to primary amine nucleophiles. Although aldehydes do not naturally occur in macromolecules of interest, they can be created from oxidizable sugar groups, typical constituents of glycosylated proteins [100]. Besides, the ribose from RNA is a reducing sugar [101]. The carbon-carbon bond between adjacent hydroxyl groups in carbohydrate sugars is cleaved (oxidized) by periodic acid (HIO_4_) from dissolved sodium periodate (NaIO_4_) to yield reactive aldehyde groups (see reaction 4 in Table 2) [56]. Carbonyls can be produced in glycoproteins and other polysaccharide-containing molecules by oxidation with sodium meta-periodate and then conjugated with hydrazide-activated cross-linkers and labeling compounds to form hydrazone bonds, stable enough for protein conjugation applications. Aldehydes created by periodate-oxidation of sugars in biological samples react with hydrazides at pH 5–7 to form hydrazone bonds (see reaction 5 Table 2) [102,103]. Hydrazide chemistry is useful for conjugating polyclonal antibodies through glycosylation sites, often located at domains away from the parotopes preserving their functionality [102]. Alkoxyamine compounds conjugate with carbonyls (like hydrazides) to create an oxime linkage and can also use aniline as a catalyst [103] (see reaction 6 in Table 2). 

Reductive amination is a zero-length cross-linking method where the electrophilic carbon atom of an aldehyde interacts with a primary amine nucleophilic nitrogen to yield a weak Schiff base, which may rapidly hydrolyze in aqueous solutions. Sodium cyanoborohydride (NaCNBH_3_) is a mild reducing agent that reduces the Schiff base to a secondary stable alkylamine linkage without reducing other chemical groups in the ligands [56,104] (see reaction 7 in Table 2). 

#### 5.4.3. Amine (R-NH_2_)

Primary amines exist at both the N-terminus of each polypeptide chain and in the side chain of lysine residues. Thanks to its positive charge at physiologic conditions, primary amines are usually on the outermost protein’s surface, more accessible for conjugation without denaturation. Many reactive chemical groups target primary amines, including N-hydroxysuccinimide ester, imido ester (the most common), carbodiimide, iso and iso(thio)cyanate, sulfonyl chloride, anhydride, aldehyde, carbonate, acryl azide, fluoro benzene, fluorophenyl ester, and epoxide [77]. EDC activation of carboxylate molecules forms NHS reactive activated esters prone to react with primary amines in slightly alkaline conditions, yielding stable amide bonds (see rection 8 in Table 2). Yet, NHS ester hydrolysis competes with the primary amine reaction. The hydrolysis rate is faster with less efficient cross-linking as the buffer pH is high and protein concentration lower. The hydrolysis half-life is 4-to-5 h at pH 7.0 and 0 °C but 10 min at pH 8.6 and 4 °C, which can be monitored at 260 to 280 nm (absorption wavelength of NHS) in a primary amine-free solution. Sulfo-NHS esters have a charged group that increases the water solubility of cross-linkers containing them and prevents sulfo-NHS cross-linkers from permeating the cell membrane with no effect on the reaction chemistry [86,105]. 

Imidoester cross-linkers interact with primary amines to form protonated amidine bonds, having a positive charge at physiological pH [106,107,108]. They have been used to study protein structure and molecular associations in membranes and immobilize proteins onto NPs while preserving the pI of the native protein (see reaction 9 in Table 2). Such amidine bonds are reversible at high pH when the more stable and efficient NHS ester cross-linkers are preferred. The half-life and reactivity with amines increase as the pH is high and therefore, the cross-linking is more efficient [56]. Maleimide is used combined with amine-reactive NHS ester chemistry (heterobifunctional crosslinkers) to enable controlled, two-step conjugation of purified peptides and/or proteins at near-neutral conditions (pH 6.5–7.5), forming stable thioether linkages. Since maleimide-activated crosslinkers react specifically with sulfhydryl groups (-SH), disulfide bonds in protein structures must be reduced to free thiols to react with maleimide reagents while excluding them from reaction buffers to avoid reaction competition (see reaction 10 Table 2) [106]. Yet, as TCEP does not contain thiols, it does not have been removed from these reactions.

Amino functional groups not only can be at the biomolecule structure but the surface of NPs. For instance, amine-terminated carbon dots (CDs-NH_2_) were functionalized with ampicillin, offering a new alternative for visible light-triggered antibacterial treatment [109]. Ampicillin-linked NPs were more stable in solution than the free ampicillin counterpart. The ampicillin-modified CDs-NH_2_ surface, together with the generation of moderate quantities of reactive oxygen species (ROS) under visible light illumination, demonstrated to be very effective in inactivating the growth of *Escherichia coli*. The platform is an example of combining the antibacterial activity of a drug (ampicillin) with the intrinsic theranostic properties of NPs (CDs-NH_2_). 

#### 5.4.4. Sulfhydryl (R-SH)

It is part of cysteine’s side chain, which commonly joins their side chains via disulfide bonds as part of a protein’s secondary or tertiary structure. The disulfide must be reduced to sulfhydryls to make it reactive towards maleimides, haloacetyls and pyridyl disulfides to cross-link to form stable thioether linkages near-neutral conditions [56]. Maleimide chemistry is used combined with amine-reactive NHS ester chemistry through heterobifunctional cross-linkers to enable controlled, two-step conjugations, free-thiols can quench maleimide excess and ethylenediaminetetraacetic acid (EDTA) can chelate stray divalent metals that promote non-reactive sulfhydryls’ oxidation [108,110,111,112]. Most haloacetyl cross-linkers contain iodoacetyl or bromoacetyl groups that react with sulfhydryl groups by nucleophilic substitution of iodine (or bromine) with a sulfur atom from a sulfhydryl group resulting in stable thioether linkages [83]. Limiting free-halogen generation is desirable to avoid cross-reactions with tyrosine, histidine and tryptophan residues (see reaction 11 in Table 2). Just a slight excess of the iodoacetyl over sulfhydryl groups at pH 8.3 would ensure sulfhydryl selectivity, but without free-sulfhydryls or with a large excess of iodoacetyl group, the iodoacetyl group can react with other amino acids [113]. Pyridyl disulfides also react with sulfhydryl groups by exchanging the -SH group to form disulfide bonds, cleaved with disulfide reducing agents, such as DTT and releasing 2-pyridyldithiol [114] (see reaction 12 in Table 2). 

One typical example in this category is the chemisorption of a thiolated photosensitive structure to AuNPs or Au nanorods (AuNRs). Photo-triggered micelles from the chitosan natural base polymer were functionalized with thiourea and then modified by grafting poly(L-lactide), poly(N-isopropylacrylamide), and poly(acrylamide) to form thermo-sensitive micelles [82]. Whereas AuNRs were chemisorbed at the micelle’s outermost surface, inducing photosensitivity to the nanocarrier, NIR light exposure simultaneously created local heat by AuNRs, shrinkage the micelles and drug release. Of the loaded paclitaxel, 38% was released at MCF7 cells after exposure to NIR light. Recent advances in the applications of AuNps for drug and gene delivery have been reviewed elsewhere [115]. These works well-illustrated, stable, versatile, and attractive AuNP-based scaffolds assembled by different monolayer structures that allow straightforward drug conjugation and tunning properties. The AuNP-conjugated structures can be intracellularly released by glutathione through the thiol functionality and an external light stimulus. 

#### 5.4.5. Photo-Reactive

Although photo-reactive groups are associated with unfavorable NP properties such as poor absorption, low aqueous solubility, and swift metabolization, they are used more and more in novel formulations [23]. The two most common photo-reactive chemical groups are diazirines and aryl-azides, which are activated by exposure to UV or visible light, most often used as heterobifunctional cross-linkers and widely used for nonspecific bioconjugation in vitro and in vivo. When an aryl azide is exposed to UV-light it forms a nitrene group that can initiate either an addition reaction with double bonds, an insertion into C–H and N–H sites, or subsequent ring expansion to react with a nucleophile; this is the reaction path that dominates in the presence of primary amines [1,26] (see reaction 13 in Table 2). Diazirines have better photostability and are more rapidly and efficiently activated with long-wave UV light than with phenyl azides by generating reactive carbene intermediates, which form covalent bonds with any amino acid side chain or peptide backbone [22] (see reaction 14 in Table 2). 

As commented, the azobenzene photo-reactive group was chemically introduced into a chitosan backbone and LRNs formed by nanoprecipitation. The resultant LRNs were functionalized with a FITC-CTB for the enhanced uptake by cardiomyocyte cells. The photoactive groups suffer a reversible isomerization from cis to trans by UV absorption and trans to cis by visible light, destabilizing the nanostructures, thus releasing the cargo into the cells [99]. Another interesting approach was a photoinduced scission of azo-modified polymer brushes covalently linked to a solid surface [116]. Photosensitive polymer brushes were prepared through analogous polymer attachment of azobenzene groups to surface-attached poly(methacrylic acid) (PMAA) chains to form surface relief gratings. The photosensitive brushes’ topography regarding the grating stripes’ height was controlled by adjusting the UV irradiation time.

#### 5.4.6. Azide (R-N_3_)

Azide and phosphine groups recognize each other to produce an aza-ylide intermediate trapped to form a stable covalent bond with high specificity in typical samples but not natural or endogenous biomolecules. This cross-linking method called the Staudinger reaction depends on a pair of unique reactive groups specific to one another but foreign to biological systems [54,117]. Because they do not naturally occur in cells, they may react only in biological samples with minimal background, within the “chemoselectivity” concept. See reaction 15 in Table 2. 

A 4-azido-2,3,5,6-tetrafluorobenzyl methacrylate (ABMA) homopolymer was recently modified with phosphines after polymerization to give stable iminophosphoranes and prepare well-defined spherical, worm-shaped, vesicular NP gels with azide-functional cores that underwent a thermally reversible degelation [118,119]. Multicomponent modification of NPs with phenylacetaldehyde, morpholine, piperidine, or the *N*,*N*′-dimethylethylene cross-linker led to the corresponding amidine derivatives (core modification) in one step. UV irradiation of NPs led to their stable cross-linking through the formation of reactive nitrene intermediates, avoided disassembly in nonselective solvents, resulting in a simple and reagent-free cross-linking strategy azide-polymer chemistry [120].

#### 5.4.7. Hydroxyl (R-OH)

The hydroxyl groups’ chemistry is essential for functionalizing polysaccharides, glycoproteins, sugar of nucleic acids, or polymers as PEG. Like the amide bond formation, the direct coupling of carboxylic acids to alcohols requires activation for the ester bond formation. Many of the methods mentioned for activating carboxylic acids for amide bond formation, such as those based on acyl halides, acyl imidazoles and carbodiimides/O-acylisoureas, achieved with *N*,*N*′-diisopropyl carbodiimide (DIC), DCC or EDC, also work in this case [102,121,122]. However, the risk of forming the non-reactive N-acyl urea is higher as alcohols are poorer nucleophiles than amines. Additives such as 4-dimethylamino pyridine (DMAP), 1-hydroxy benzotriazole (HOBt) or 7-aza-1-oxy-l,2,3-benzotriazole (HOAt) [123,124] minimize this side reaction. The corresponding activated intermediates rapidly react with the alcohol to form the desired ester in a high yield. Another alternative is the ester formation via alcoholysis of mixed anhydrides in the presence of an organic base [125]. The alcohol also can be activated toward nucleophilic attack from the carboxylic acid (Mitsunobu reaction) by reacting with a phosphine and dialkyl azodicarboxylate (see reaction 16 Table 2). Hydroxyl groups can indirectly react with amines giving stable carbamate bonds [122] with 1,1′-carbonyldiimidazole (CDI), DSC and *N*-hydroxysuccinimidyl chloroformate (HSC). CDI can react with hydroxyl groups generating an imidazolyl carbamate active intermediate that reacts with the desired amine via a stable urethane linkage and releasing an imidazole molecule. DSC and HSC can also be used in nonaqueous environments as they rapidly hydrolyze. 

A phthalocyanine-loaded nanostructured lipid carrier was functionalized with FA and tested as targeted photodynamic therapy (PDT) against breast cancer [126]. For the nanocarrier assembly, the PF127 polymer was first esterified with FA by the CDI coupling chemistry to ensure that at least one or more of the two hydroxyl groups of PF127 were conjugated to carboxylic acid groups of FA and further emulsified with an oil phase containing the phthalocyanine. Similarly, carboxylic acid-hydroxyl coupling through CDI has been explored for synthesizing FA–PF127 magnetic NP clusters for imaging and theranostic applications [119]. Conjugation of α-, β-, and γ-cyclodextrins (CD) to graphene oxide (GO) sheets was achieved by the esterification of carboxyl functional groups of the GO with hydroxyl groups of CD, catalyzed by EDC/DMAP. The reaction produced stable and biocompatible nanocarriers to encapsulate SN38, an active metabolite of irinotecan approved for clinical use in metastatic colorectal cancer, to overcome its solubility and stability issues and reduce its side effects [127]. The synergistic treatment platform was demonstrated to have the potential for colorectal cancer therapeutics. 

#### 5.4.8. Silanization

It is the process of covering, e.g., a metal oxide surface with organofunctional alkoxysilane molecules by linking its hydroxyl groups to the alkoxy groups on the silane, thus forming a covalent -Si-O-Si- bond. The purpose is to form bonds across the mineral-organic components interface when assembling hybrid nanobioconjugate drug delivery systems. This method commonly combines the power of targeted delivery with the spatiotemporal control of light activation [128]. 

A cell-impermeable fluorescent compound delivered exclusively to the cytosol of multidrug-resistant cancer cells based on light-activated disruption of intracellular vesicles after uptake was proposed [87]. The authors developed a synthesis procedure based on silanization of mesoporous silica NPs with tetraethyl orthosilicate (TEOS), mercaptopropyltrimethoxysilane (MPTMS), and Pluronic F-127 as a secondary surfactant to obtain accurate size control and improve targeting efficiency. 

#### 5.4.9. Bioaffinity Interactions

They are interactions based on the affinity of biomolecules. For example, biotin (Vitamin B_7_, vitamin H) has a strong affinity for avidin, neutravidin and streptavidin. Biotin can bind the tetrameric avidin rapidly, with high affinity and specificity in a broad range of pHs and temperatures, forming the strongest non-covalent bonding known in nature with a dissociation constant Kd of 10^−15^ M [77,129,130]. Avidin is 66–69 KDa in size, and 10% of its composition is carbohydrates. Its pI of 10 makes it is mainly positively charged in conjugation reactions with pseudo-catalytic activity and has a high tendency to aggregation. Neutravidin is the deglycosylated version avidin of 60 KDa and a near-neutral pI (pH 6.3), minimizing nonspecific interactions with DNA/RNA or the negatively charged cell surface to circumvent the mentioned issues [4,131]. By removing the carbohydrates, lectin binding minimizes while biotin-binding affinity retains; besides, neutravidin still has lysine residues that remain available for derivatization or conjugation with a strong affinity for biotin (Kd = 10^−15^ M). In biochemical applications, streptavidin pI (pH 5), which binds very tightly to biotin (Kd = 10^−14^ M), may be used interchangeably with neutravidin. Streptavidin is a homotetrameric protein of 52.8 KDa extracted from the bacterium *Streptomyces avidinii*, highly resistant to organic solvents and denaturants detergents, proteolytic enzymes, and extreme temperatures and pHs [52]. The mentioned strength and specificity made such interactions applicable to graft plenty of biomolecules to nanocarriers and colloidal particles. The surface of live wild-type LN-229 cells was biotinylated to test a streptavidin-NP conjugate-based delivery strategy to multidrug-resistant cells by bioaffinity interactions, as will be detailed in the end of this section. It was assessed through targeting expressed P-glycoprotein bioreceptor in the cells following antibody staining [87].

#### 5.4.10. Layer-by-Layer (LbL) Assembly

LbL assembly is a versatile method for thin film deposition and coating substrates with polymers, colloids, biomolecules, and even cells in a precisely controlled manner [132,133]. Control of drug release can be achieved by designing formulations containing alternating layers of the same or different polymers of opposite charge with the drug (or multiple drugs) loaded between layers and washing steps between the processes. This is a simple manner of controlling and sustaining the same or multiple drugs’ release, depending on the expected therapeutic application [134]. Layers are commonly assembled from polymers of opposite charges by enthalpic or entropic driving forces. For example, polystyrene sulfonate (PSS) of negative charge can be intercalated between polyallylamine hydrochloride (PAH), polyethyleneimine (PEI), or polyacrylic acid (PAA) of positive charge. Chitosan is a natural polymer of positive charge widely used in LbL arrangements. LbL offers the opportunity to self-assemble a wide variety of appropriate building blocks and has other merits such as low process temperature, molecular resolution composition and thickness control [133,135].

A zinc phthalocyanine was loaded onto gelatin NPs functionalized with PSS/PAH polyelectrolytes by LbL assembly [136]. Changes in the ζ-potential indicated successful alternating decoration of the polyanion PSS and polycation PAH directly on the gelatin NPs. The LbL deposited polyelectrolyte bilayer was very efficient in reducing the release rate and alleviating the initial burst for drugs loaded in the gelatin NPs. Cell viability of a mouse macrophage carcinoma line J774 A-1 decreased with light dose, demonstrating the photobiological activity of the functional NPs and their potential for PDT use [137]. In the same context, hyaluronic acid-functionalized chitosan NPs loaded with oxaliplatin were encapsulated into Eudragit S100, administered orally, and tested for effective delivery to colon tumors [138]. The systems showed relatively high localized drug concentration with prolonged exposure time and the potential to lower systemic toxicity and enhance antitumor efficacy for colon cancer treatment. 

**Figure 5:**
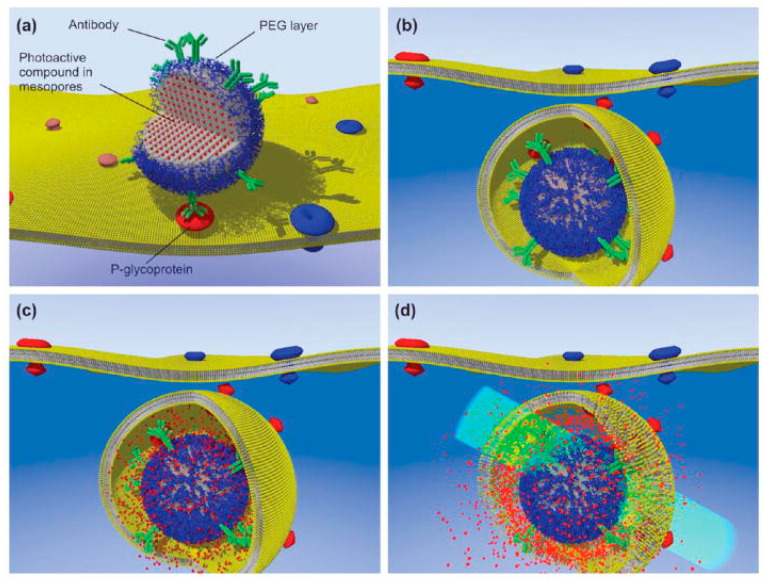
Schematic of light-activated and targeted cytosolic delivery of membrane-impermeable compounds. (**a**) Antibody-functionalized NPs are loaded with fluorescent Alexa546 model dye and targeted to cells expressing P-gp-green fluorescent protein (GFP bound to the P-glycoprotein transporter). After NPs endocytosis (**b**), the cargo was released in the endosome (**c**). Exposure to light at the dye’s excitation wavelength (546 nm) promoted ROS-mediated membrane damage (**d**) with cytosolic delivery of Alexa546 exclusively in the P-gp expressing cells. Reprinted with permission from [87]. Copyright 2010 American Chemical Society.

To close this section, it is worth commenting on an example that illustrates many of the functionalization strategies described herein. It is a cell-impermeable fluorescent compound delivered exclusively to the cytosol of multidrug-resistant cancer cells expressing a glycoprotein in a mixed population based on light-activated disruption of intracellular vesicles after internalization of biofunctionalized mesoporous silica NPs loaded with cargo [87]. Silanization of mesoporous silica NPs with TEOS, MPTMS and PF-127 lead to accurate size control and improved targeting efficiency. The NPs were functionalized by covalent attachment of streptavidin onto their surface via a heterobifunctional cross-linker with NHS-PEG-maleimide (long arm) or with LC-SMCC cross-linkers (shorter arm) [128]. NPs were covalently linked to Alexa546-maleimide through the free sulfhydryl groups, saturating the silica NPs with hydrolyzed dye. The surface of live LN-229 cells was biotinylated to test cell surface attachment to streptavidin-functionalized NPs by bioaffinity interactions. Cytosolic delivery of a 3 kDa dextran-FITC conjugate was also evaluated. Release into the cytosol was observed after short exposure to green excitation light. Proteins such as neutravidin could also be delivered to the cell cytosol with slower diffusion kinetics than a dye molecule. The streptavidin-NP conjugate-based delivery strategy was assessed through targeting expressed P-glycoprotein bioreceptor in wild-type LN-229 cells following antibody staining by taking advantage of the bioafinity interactions [87]. It was observed that bioconjugation with a long arm cross-linker maximized the targetability of the NPs. Overall, the approach promises to expand the pharmacological arsenal to cell-impermeable compounds to overcome multidrug resistance (Figure 5).

## 6. Characterization of Photosensitive Functional Nanocarriers

### 6.1. Physicochemical Properties

Functionalized NPs can be characterized by a wealth of optical and physical methods. The distinct optical and physical properties of NPs are essentially due to their different composition, size, shape, morphology, colloidal stability, and linker properties. Thereby, adequate reliable and comprehensive characterization with multiple techniques is essential to study the NPs and quality control of the fabrication process, which is typically recommended to avoid misleading interpretations. The methods to evaluate the physicochemical properties of nanocarriers have been extensively reviewed elsewhere. Therefore, Table 3 summarizes the main physicochemical methods to characterize LRNs.

### 6.2. Cargo Release

Light may trigger changes in the physical properties of some surfaces, such as wettability, hydrophobicity, lubrication adhesiveness and conformational changes, of interest in biotribology, controlled drug release, and cell growth and separation, among other fields [14]. Particularly in drug release, such light-triggered changes have a profound impact on the release kinetics. Kumari et al. described five release mechanisms (i) desorption of drug bound to the surface, (ii) diffusion through the excipient matrix, (iii) diffusion through the polymer/lipid wall, (iv) erosion of the matrix of NPs and (v) a combined process of diffusion erosion [152]. The photoresponsive properties and photo-triggered cargo release capabilities of NPs are dependent on their absorption cross-section (a measure of the probability of photon absorbance by each particle) and quantum yield [1]. 

### 6.3. Photoisomerization Properties

Photoisomerization of molecules, e.g., azobenzene chromophore, may produce substantial changes in molecular geometry and surface free energy, giving rise to reversible switching of dipole moment and wettability. The transazobenzenes typically have small dipole moments, exhibit hydrophobic properties, and isomerize upon irradiation with UV light to the cis configuration, which possesses a higher dipole moment and decreases hydrophobicity [28]. Due to the light effect, UV-visible (UV-Vis) spectroscopy is one of the reasonably standard methods for characterizing such systems [13,153]. The product of the absorption cross-section and quantum yield determines the efficiency of this energy conversion process. A single UV-Vis photon possesses sufficient energy to achieve photochemical reactions that have been widely used as the basis for photoresponsive NPs [14]. In this context, simple irradiation with a hand-held UV lamp emitting at different wavelengths had been strategic to stimulate photoresponse and release drugs from NPs. Yet, some particular characterization is necessary for this kind of NPs, e.g., two-photon microscopy (TPM) that utilizes two NIR photons as the excitation source. In this methodology, two photons of approximately twice the wavelength of single-photon excitation stimulate the fluorophore to the excited state. The emission process happens in the same way as with the one-photon excitation.

### 6.4. Fluorescent Properties

Fluorescence is advantageous because it provides higher spatial resolution using excitation in the NIR spectral window [154]. Innovative techniques have been reported to stimulate photosensitive NPs, e.g., emission depletion microscopy (STED) uses a second laser to suppress the fluorescence emission from the fluorophores located off the excitation center. When an excited-state fluorophore encounters a photon that matches the excited-ground state energy difference, it can be brought back to the ground state through stimulated emission before spontaneous fluorescence emission occurs. This process effectively depletes excited-state fluorophores capable of fluorescence emission. Especially designed architectures belonging to coumarin and SP classes have been used with great success in this type of microscopy [75,85,116]. 

### 6.5. Surface Coverage and Functional Ligands

Determination of the surface coverage of functional ligands is another determinant parameter in functionalized-NPs characterization. The quantification techniques and methods developed for characterizing the shell thickness, the ligand orientation, and the number of active moieties on NPs are briefly summarized below [13,33,39]. DLS and differential centrifugal sedimentation are some direct techniques based on measuring the particle diameter and the protein shell thickness around NPs [14,155]. The RMM has been reported for the direct quantitation of immobilized proteins. Some authors used XPS, a technique that measures the elemental composition and allows to infer their bonding state and estimate the overlayer structures, thickness, and surface chemistry of NPs [156]. The quantification of protein is also relevant, which methods are based on Lowry-, Bradford-, and bicinchoninic acid-assays [157]. 

Electrophoresis provides a method for characterization of bare and biofunctionalized NPs as when an electric field is applied, their migration through the gel depends on their shape, size, and charge [143,158]. According to their electrophoretic mobility, polyacrylamide gel electrophoresis (PAGE) is one of the most useful methods to separate biomolecules, such as proteins and peptides. Thus when the molecule used to functionalize the NPs is a peptide or a protein, this method is an indirect way to analyze the efficacy of the process. Other proper methodologies are based on dot-blot immunoassays and enzyme-linked immunosorbent assay (ELISA). The only requirement is to have a detection antibody able to detect the biomolecule selected for the functionalization. Besides, it could also give information about the biomolecule orientation once it is attached to the NP surface, as mentioned.

## 7. Biomedical Applications of Photo-Controlled Drug Delivery Systems

### 7.1. Biocompatibility and Biodegradability

Nonbiodegradable polymers show limited clearance in biological conditions, so an exhaustive optimization is necessary to advance biomedical applications [159]. In contrast, biodegradable polymers offer outstanding opportunities in this field related to their high drug loading capacity, encapsulation efficiency and facile modification, and especially to their biocompatibility, control over biodistribution, and degradation kinetics [14]. Photocleavable species are conjugated to the polymer side chains as protecting groups of nucleophiles, such as alcohols, thiols, and amines, to improve the biocompatibility of photosensitive polymers [25,33]. Upon light irradiation, photocleavage of the light-responsive units leads to the deprotection of these functional groups, initiating a consecutive degradation process of polymer main chains and thus releasing the payload of polymeric NPs. For example, utilizing the photoinduced hydrophobic-to-hydrophilic property of hydrophobic SP isomerized to the hydrophilic MC, Wang et al. reported a nanogel of poly(acrylic acid-co-spiropyran methacrylate) cross-linked by *N*,*N*-bis(acryloyl)cystamine. In vitro cytotoxicity assays revealed that the empty nanogels possessed good biocompatibility before and after UV light irradiation (360 nm, 15 mW cm^−2^, 1 min) even at high concentrations (50 μg mL^−1^) [160]. Apart from SP, currently, many molecules with precise alterable spatiotemporal structures, such as chiral helicene, azobenzene, diarylethene, and binaphthyl compounds, have been explored as photoswitches [1,3]. The drug release processes and their photocage-dependent biodegradability, e.g., the light-responsive units’ photocleavage, lead to the deprotection of these functional groups, which can initiate a consecutive degradation of polymer main chains and release the polymer nanoparticles’ payload [26].

### 7.2. Functional Nanocarriers

The increased use of functionalized polymeric NPs is because of their properties as intelligent systems, target specificity that increases efficiency and efficacy of therapeutic regimes while reducing side effects. Until a few years ago, it has been difficult to treat certain diseases like cancer, which required target-specific action [161]. However, although the light is an external stimulus that can be applied directly to the LRNs to release cargo with good spatiotemporal control, it possesses some limitations related to its specificity. Moreover, all light-activated systems suffer from limited tissue penetration and UV irradiation can only be applied to very superficial parts of the body, such as the skin, mucosa, and eyes. Besides, a light stimulus would be more effective if the targeted tissues are distinguished from the healthy ones. Therefore, the idea of modifying LRNs with different moieties and ligands search for site-specific cargo delivery imparting stealth effects and/or eliciting specific cellular interactions to improve the nanosystems’ safety and efficacy [162,163].

One of the first approaches to using photosensitive compounds was their coupling to biomolecules, which allowed their properties to change against light stimulation. These biomolecules underwent predictable folding or unfolding in a biological context. For instance, if the light is used for disruption, biomolecules functionalized with azobenzene such as deoxyribonucleic acid and peptides can undergo substantial conformational changes upon isomerization, leading to designed and reproducible changes in bio-material function [164,165]. Asanuma et al. reported for the first time the concept of chemical incorporation of an azobenzene derivative that could modulate the melting temperature of DNA throughout the double helix disruption. As a proof-of-concept, a NP whose surface was decorated with a peptide ligand (YIGSR) and inactivated by caging with 4,5- dimethoxy-2-nitrobenzyl (DMNB) photocleavable group demonstrated phototargeting. In the absence of light, the NPs did not bind to cells, but when irradiated at 365 nm, the DMNB group was cleaved from the YIGSR, allowing the NPs to bind to cells bearing integrin 1, the target for YIGSR. Since integrin 1 is widely distributed on many cell types throughout the body, this approach could, in theory, allow NPs to bind specifically to any tissue that can be irradiated. This approach also has the advantage over conventional ligand-targeted monotherapies of not requiring a tissue-specific ligand or even not requiring knowledge of a tissue-specific disease marker [153].

Despite the versatility of LRNs to fight diseases, most of the work carried out focuses on cancer, so we will focus on the findings on this pathology and ultimately the use of functionalized LRNs for other pathologies classified according to the targeting biomolecule as follows.

#### 7.2.1. DNA-ARN Functionalized NPs

Cheng et al. [166] successfully developed a dual light- and temperature-responsive end-capped poly(propylene) glycol (BU-PPG) polymer that spontaneously self-assembled to form micelle-shaped NPs in phosphate-buffered saline (PBS) via supramolecular interactions among uracil moieties. Functionalization was achieved using an aza-Michael addition reaction in the presence of potassium, invoking cooperative assembly of the BU-PPG upon association with the nucleobase adenine derivative methyl 3-(6-amino-9*H*-purin-9-yl) propanoate (A-MA). To proof-of-concept of phototargeting, an increase in temperature above NPs significantly accelerated the release of A-MA from irradiated micelles due to the destruction of the hydrogen bonds between the A-U base pairs. These findings further suggest that the introduction of complementary interactions and photosensitive uracil moieties into water-soluble supramolecular structures could be employed to safely deliver nucleobase-complementary molecular drugs to the slightly higher temperature microenvironment of solid tumors and effectively minimize the premature release of the A-MA at average physiological temperature. 

Kang et al., 2011 [167] described a photoresponsive DNA-crosslinked hydrogel development, whereas photosensitive azobenzene moieties were incorporated into DNA strands as crosslinkers photoregulated by two wavelengths with a reversible sol-gel conversion. A photoresponsive azo-incorporated DNA crosslinker was synthesized individually by photo-initiated polymerization of 5′ acrydite-modified oligonucleotide monomer mixed with acrylamide. This LRN was used for precisely controllable encapsulation and release by irradiation at 450 nm the chemotherapy drug doxorubicin in an in vitro model. The results showed a net release rate of 65% within 10 min, while the released drug maintained its therapeutic effect. This hydrogel system provides a promising platform for drug delivery in targeted therapy. 

#### 7.2.2. Peptides and Proteins

The active targeting is achieved by molecular recognition of the targeted cells/tissues by various signature molecules overexpressed at the diseased site via the ligand-receptor, antigen-antibody interactions being peptides and proteins widely explored for functionalization of NPs. Biomarkers preferentially expressed on newly formed blood vessels in tumors, such as the vascular endothelial growth factor (VEGF) and the apha(v)beta(3)integrin (αvβ3), are also common. Among biomarkers, the integrin ligands have been widely studied. For example, the Arg-Gly-Asp (RGD) is a tripeptide that can mimic cell adhesion proteins and bind to cell-surface receptors [130]. Peptides in connection to hydrogels offer alternatives for the design of LRNs [168,169]. By modifying agarose hydrogels with photosensitive S-2- nitrobenzyl-cysteine, selectively removed the desired region by high-intensity light from a UV laser, releasing the reactive sulphydryl groups. Maleimide terminated biomolecules were patterned within the functionalized agarose gel channels by reacting with free sulphydryl groups. RGD peptides were immobilized within the exposed region as demonstrated by selective spreading and migration of dorsal root ganglia cells. This strategy enabled the formation of spatially selective peptide-modified microstructures within hydrogel matrices. 

CPPs can be used for the intracellular delivery of a wide variety of cargoes. Shamay et al. [55] showed that modification of the CPP cationic residues with photolabile caging molecules that neutralize its charges would lead to conditional light-dependent cell-penetrating functionality. They described two carrier systems for dye and drug-delivery, based on the N-(2-hydroxypropyl) methacrylamide (HPMA) copolymer scaffold that could be selectively transported into cancer cells following light-dependent uncaging of the caged CPP. An amine-terminated cCPP was coupled to a FITC-labeled HPMA copolymer precursor having active p-nitrophenyl ester groups. Once illuminated by UV light, these protecting groups were cleaved, the positively charged CPP regained its activity and facilitated rapid intracellular delivery of the polymer-dye or polymer-drug conjugates into cancer cells. 

Three-dimensional biomolecular patterning within hydrogels can also provide a biomimetic environment for in vitro cell culture. Shoichet et al. [81,170] created 3D amine patterns within an agarose hydrogel by chemically bonding it with 6-bromo-7-hydroxymethyl coumarin protected amine, which was subsequently uncaged using a two-photon laser [170]. Proteins have also been patterned within gels using similar methods. For example, immobilization of fibroblast growth factor-2 (FGF2) was achieved using two synthetic schemes. One approach was to form disulfide bonds between FGF2 and agarose gels directly by uncaging coumarin-4-yl-methyl protected thiol groups. An alternative strategy was to use a two-step binding procedure in which maleimide-modified human serum albumin (HSA) was immobilized and reacted with an albumin-binding domain fusion protein (FGF2) [170]. 

Han et al., 2015 [171] developed a complex structure, a pH-sensitive chimeric peptide Fmoc-12-aminododecanoic acid-H8 R8 -PLGVR-PEG8 to co-deliver a protoporphyrin IX (PpIX) photosensitizer and a DNA plasmid simultaneously. At physiological pH and in the presence of matrix metalloproteinase-2, the hydrolysis of a peptide Pro-Leu-Gly-Val-Arg-NH2 (PLGVR) sequence and exfoliation of PEG resulted in uptake of the carrier by tumor cells (rich in matrix metalloproteinase-2). The peptides were synthesized using the standard fluorenylmethyloxycarbonyl solid-phase synthesis method, and PpIX loaded chimeric peptide was prepared via a solvent evaporation method. This uptake was followed by two-step light irradiation: (1) short-term illumination for endosomal escape due to the proton-sponge effect of H8 and the photochemical internalization PCI effect of PpIX aimed at improving plasmid DNA expression, and (2) long-term irradiation to activate the phototoxicity of PpIX. It was concluded that the dual step light therapy could address the bottlenecks of synergistic treatment such as endosomal escape, interference among antitumor drug toxicities, cell bioactivity, and gene transfection. 

Compared with enzyme-sensitive cleaving mechanisms, the specificity and efficiency of a photo-cleavable group offer modular chemical approaches for rationally designing selective cell-penetrating nanostructure-based new therapeutic agent delivery systems from photo- and pH-responsive polypeptides (PPPs), which transport cargos more efficiently to target tumor cells [3]. The PPP system includes a CPP sequence (CGRRMKWKK), a photo-decomposable group (4,5-dimethoxy-2-nitrobenzyl group) and a pH-sensitive inhibitory peptide (EEEERRRR). CGRRMKWKK is a CPP derived from Penetratin that can enhance membrane translocation efficiency. The cell penetration ability of CPPs was effectively shielded by the opposite electric charges within the pH-sensitive inhibitory peptides in circulation. Upon NIR irradiation at the tumor position, the photosensitive group was cleaved, while the pH-sensitive inhibitory peptide eliminated the electrostatic attraction at a lowered pH simultaneously. After cleaving the linker and eliminating the electrostatic attraction, the PPP could release its inhibitory peptides to expose the CPPs [172].

#### 7.2.3. Antibodies

**Figure 6:**
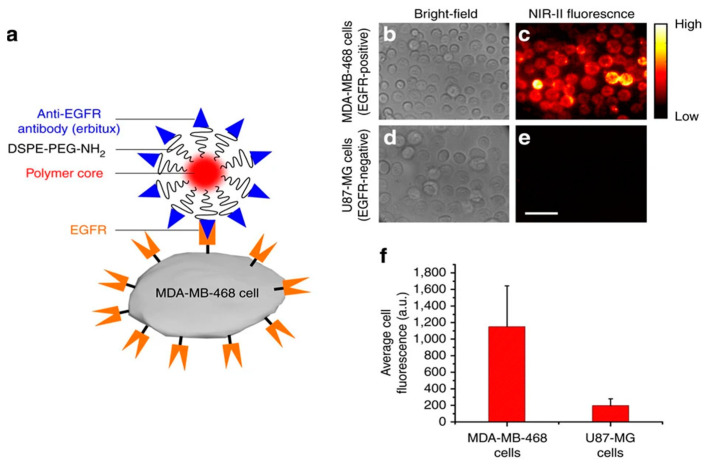
(**a**) Schematic illustration showing the structure of pDA-PEG-Erbitux bioconjugate, where the anti-EGFR antibody (Erbitux) selectively targets EGFR on the cell membrane of an MDA-MB-468 cell. White-light (**b**) and NIR-II (**c**) fluorescence images of EGFR-positive MDA-MB-468 cells incubated with the pDA-PEG-Erbitux bioconjugate, showing positive staining of cells. White-light (**d**) and NIR-II (**e**) fluorescence images of EGFR-negative U87-MG cells incubated with the pDA-PEG-Erbitux bioconjugate, without evident staining of the cells. The scale bar is 40 μm. (**f**) Average NIR-II fluorescence of EGFR-positive MDA-MB-468 cells and negative U87-MG cells, showing a positive/negative ratio of ~5.8. The error bars in f are the s.d. of average fluorescence intensity from 20 cells in each NIR-II fluorescence image. Reprinted with permission from [173]. Copyright 2014 Springer Nature.

The antibodies represent the most efficient ligands due to their high affinities and ability to recognize a specific part of their target. Hong et al. assembled a LRN from fluorescent copolymer, poly(benzo[1,2-b:3,4-b′] difuran-*alt*-fluorothieno-[3,4-b] thiophene) (named ‘pDA’) and phospholipid–PEG [173]. The obtained pDA-PEG NPs were successfully used as a fluorescent label for molecular imaging of cancer cells at N1000 nm by functionalizing with Cetuximab (Erbitux) for targeting EGFRs on the cell membranes of EGFR-positive breast tumor MDA-MB-468 cells. Cetuximab antibodies were then thiolated and conjugated to pDA-PEG-NH_2_ via standard crosslinking reaction between the -NH_2_ groups on the polymer and -SH groups on the thiolated antibody. More importantly, the high quantum yield of the pDA-PEG NPs affords in vivo, deep-tissue, and ultrafast imaging of mouse arterial blood flow (Figure 6).

Multifunctional targeted polymeric (NPsT) and the combination of photothermal therapy (PTT) and targeted chemotherapy can produce much more significant cytotoxicity than chemotherapy. NPsT loaded with indocyanine green (ICG) and doxorubicin (DOX) for the targeted photoacoustic imaging and photothermal ablation of oral cancer cells were developed. The chemokine SDF-1, a specific antibody, was conjugated to NPs by the carbodiimide method. The NPs were automatically targeted to tumor tissue in vitro and in vivo through CXCR4-SDF-1 interactions.

In vitro and in vivo experiments showed that the multifunctional NPs had excellent photoacoustic imaging characteristics and PTT capabilities. The photothermal material heated rapidly after laser irradiation, and the resulting heat increased cell metabolism and membrane permeability, which increased cellular NP uptake. The thermal energy generated by the heating of the material by laser irradiation and the DOX released after the phase change in the material exerted a dual killing effect on tumor cells. In vivo experiments showed that laser irradiated NPs could effectively inhibit the growth of cancer cells and that targeted NPs could accumulate in tumor tissues for a long time [174]. This photothermal NP is a flexible platform that can be readily adapted to, e.g., SARS-CoV-2 antibodies and extend to novel therapeutic proteins. Recently, it was incorporated neutralizing antibodies conjugated on the surface of photothermal NPs to capture and inactivate SARS-CoV-2 [175]. The photothermal NPs consist of a semiconducting polymer core and a biocompatible PEG surface decorated with neutralizing antibodies. Such NPs displayed efficient capture of SARS-CoV-2 pseudoviruses, excellent photothermal effect, and complete inhibition of viral entry into ACE2-expressing host cells via simultaneous blocking and inactivating the virus.

Due to the limitations of UV light stimulation, NIR light stimulation has become an alternative, so there are currently several polymeric NPs functionalized with peptides or proteins that use this type of stimulus to enhance their biomedical action [23]. For example, in cancer, PTT has attracted increasing attention. NIR is always used for PTT treatment with less biological toxicity and superior tissue penetration depth (e.g., more than 1 mm upon 808 nm irradiation) [3]. Recently, an anticancer platelet-based biomimetic formulation (N+R@PLTs) functionalized LRN stimulated with NIR irradiation was developed, integrating photothermal NPs (N) and immunostimulators (R) into platelets (PLTs). A block copolymer, naphthalene diimide-bithiophene derivative (NDI-BT), was designed as the photothermal material for this construction. Then, the N NPs were synthesized and imported into PLTs together with the immunostimulator R837 hydrochloride (R) to construct the engineered PLTs (N+R@PLTs). After intravenous injection, N+R@PLTs functioned as circulating sentinels in the bloodstream, having a sensitive response to vascular damage, as defects always weaken the junctions among vascular endothelial cells in the vicinity of tumor tissue. To functionalize the photothermal NPs with additional PLT responsiveness and immunogenicity, the NPs were then decorated with biotin, while CD42a protein on the PLT membrane was pretreated with avidin-labeled anti-CD42a antibody. They hypothesized that the highly specific biotin-avidin interaction would trigger the binding of NPs to anti-CD42a antibodies, promoting the uptake of the NPs into PLTs via the CD42a molecules. After irradiation with NIR, local hyperthermia resulted in acute vascular damage, which subsequently inducted an aggregation cascade to form an occlusion at the tumor vessel [176].

#### 7.2.4. Smaller Molecules

##### Polymers

Surface modification with relatively smaller molecules is another promising application for o-nitrobenzyl-based photocleavable linkers. In this embodiment, o-nitrobenzyl groups can connect functional polymer chains to substrates. Hydrophobicity protein immobilization [177] and cell adhesion [178] to substrates can be controlled by photosensitive materials that can be cleaved using external light [179]. They can be synthesized from photocleavable PEG-lipid by adding a photolabile o-nitrobenzyl linker between PEG chains and oleyl groups. Oleyl moieties bond to nonadherent cells via insertion into the cell membranes. Therefore, selective removal of oleyl groups from PEG chains prevented cell adhesion and promoted detachment from photoresponsive substrates. Micropatterned cell populations were created by selectively exposing the PEG-lipid-coated surfaces to light using a photomask. Cells aggregated in masked regions and detached in exposed regions.

A photo-activatable ternary complex consisting of multifunctional shielding material (MSM) with photosensitizer (PS)-conjugated chondroitin sulfate (CS) and PEI-based binary complexes containing EGFR-plasmid vector based-small hairpin RNAs (shRNAs) for CD44 targeted cancer therapy was developed. The CS molecule was used as a physiologically specific glycosaminoglycan composed of alternating units (N-acetylgalactosamine and glucuronic acid) conjugated to the polymer by DCC reaction, whichwasuptake by cancer cells via CD44 receptor-mediated endocytosis,. The treatment of HCT116 cells highly expressing CD44 receptors with PEI-PhA-CS-shRNA, irradiation at 633 nm and a 0.5 J/cm^2^ induced EGFR gene silencing up to ~85%. In addition, the combination of PEI-PhA-CS-shRNA and photoirradiation at 30 J/cm^2^ inhibited the tumor growth in HCT116 tumor-bearing BALB/c nude mice [180].

Ma et al. [181] used a star-shaped polymer consisting of a porphyrin core and arginine-modified 3rd generation dendron arms. A star-shaped copolymer (PP-PLLD-Arg) with a photochemical uptake effect consisting of a porphyrin (PP) core and arginine-functionalized poly(l-lysine) dendron (PLLD-Arg) arms had was used to co-deliver docetaxel (DOC) and MMP-9 shRNA plasmid for nasopharyngeal cancer therapy. The functionalization of Arg-PPLD used amide bond formation with 1-hydroxy benzotriazole (HOBt) and o-benzotriazole-*N*,*N*,*N*0,*N*0-tetramethyluronium hexafluorophosphate (HBTU). NPs could bind to hydrophobic DOC because of their amphiphilic nature and bind to anti-MMP-9 shRNA plasmid through electrostatic interactions. With a diameter of 150 nm, the complexes had better blood compatibility and lower cytotoxicity in vivo. After treating the cells with complexes, MMP-9 mRNA and protein expressions decreased by ~20% compared to those treated with PBS. After irradiation at 435 nm and 1.26 J/cm^2^, MMP-9 protein expression was about 50% lower in the cells treated with complexes. In addition, co-delivering DOC and anti-MMP-9 shRNA plasmid into the polymer complex could induce cell apoptosis effectively.

##### Bioactive Molecules

Yuan et al. [182] designed an a-type amphiphilic polymer, octadecyl-PEG(biotin)-(o-nitrobenzyl)-octadecyl chain, with a photocleavable group placed between the PEG(biotin) and the octadecyl end. The polymer formed micelles with a flower-like structure where the biotin moiety was anchored between the micelles’ hydrophobic core and hydrophilic shell. Cu-catalyzed azide-alkyne cycloaddition (CuAAC) click chemistry was utilized to connect amphiphilic polymers with photocleavable PEG(biotin). Irradiation at 365 nm resulted in the cleavage of the o-nitrobenzyl group, which promoted the migration of biotin to the micelle surface, enabling ligand-receptor-mediated targeted drug delivery in human cervical cancer cells (HeLa cells) with over-expression of biotin receptors. Folate is a B vitamin and micronutrient for humans that can bind to the folate receptor through ligand-receptor interactions [61]. The folate receptor has been studied widely as a molecular target for cancer therapy. A novel type of theranostic NP folate-receptor-targeted laser-activable PLGA NPs loaded with paclitaxel (Ptx)/indo-cyanine green (ICG)-folic acid-polyethylene glycol (PEG)-PLGA-Ptx@ICG-perfluorohexane (Pfh) uses safe and approved materials and drugs to facilitate clinical translation. With laser irradiation, highly efficient PTT can be achieved. Additionally, targeted NPs can be activated by NIR laser irradiation at a specific region, which leads to the sharp release of Ptx at areas of high folate-receptor expression and ensures a higher Ptx concentration within the tumor region, thereby leading to chemo/photothermal synergistic antitumor efficacy [183].

**Figure 7:**
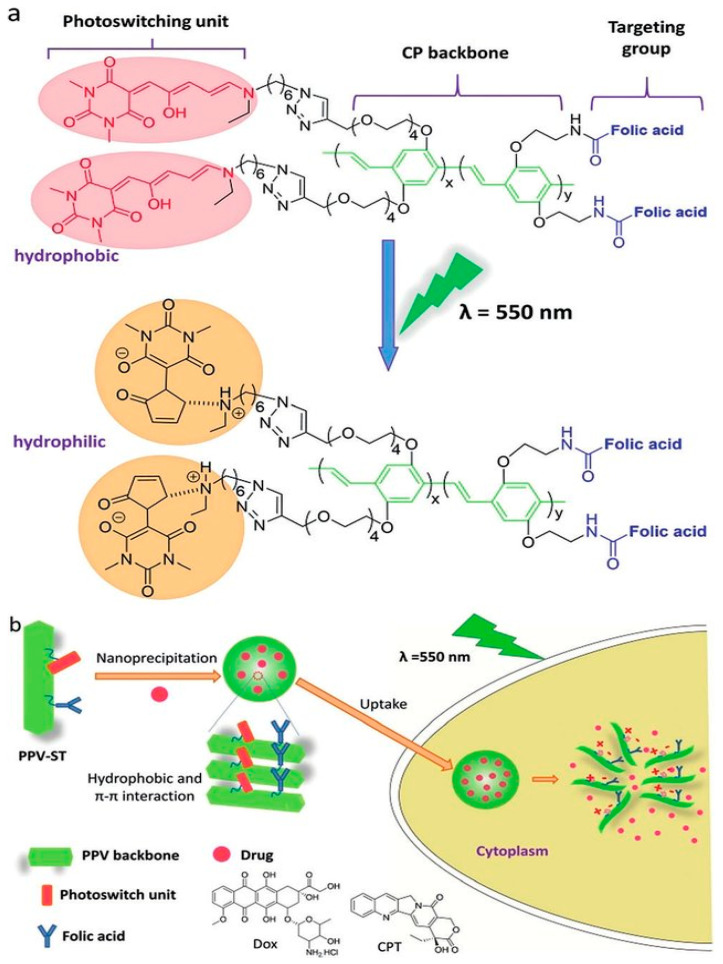
(**a**) The chemical structure of PPV-ST conjugated polymer and its structural change under visible light (λ = 550 nm) irradiation; (**b**) Schematic representation of the formation of drug-loaded PPV-ST-NP nanoparticles and structural change upon irradiation with visible light (λ = 550 nm) leading to the drug release in cells. Reprinted with permission from [184]. Copyright 2018 John Wiley and Son.

Other particles that use FA as a functionalization biomolecule were proposed by Senthilkumar based on a photo-responsive poly(p-phenylene vinylene) functionalized with donor-acceptor Stenhouse adduct (DASA) and FA units for controlled drug delivery, release and imaging. FA was conjugated by the EDC reaction [184]. Upon visible light irradiation at 550 nm, NPs underwent structure, color, and polarity changes simultaneously swell and opened-up NPs to release camptothecin (CPT) and DOX encapsulated anticancer drugs into the medium and cells. There was favorable fluorescence resonance energy transfer (FRET) from backbone to DASA units boosting the fluorescence imaging performance. Since the light triggering synchronized the structural and significant polarity changes of NPs, they showed excellent cell viability and chemical stability in dark conditions. This strategy enabled remotely controlled drug delivery and released with visible light irradiation compared with other light trigger approaches (Figure 7).

Novel and photoresponsive hyaluronate (HA) nanogel cages with programmed drug release properties and selective targetability for tumor cells upon irradiation with a UV light have been proposed. HA, a naturally occurring polysaccharide, can bind to the overexpressed CD44 receptor on the surface of various tumor cells. HA nanogels were prepared from photolabile 4-(4-(1-hydroxyethyl)-2-methoxy-5-nitrophenoxy) butyric acid (HMNB). HMNB was simply grafted to HA by using DCC and DMAP as catalysts. Photoactivation allowed accelerated DOX drug release from uncaged nanogels, improving KB tumor-cell-killing efficacy when this system was associated with local light irradiation [185].

##### Sugars

Finally, compared with common antigen-antibody recognition, multivalent sugar-protein binding has received much attention to fabricating targeted LRNs, having lower cost and lower immunogenicity. A third-generation propargyl focal point poly (amidoamine) dendron (D3) was used to afford the dendritic amphiphile D3-poly(ε-caprolactone) (PCL) with multiple NIR-sensitive diazonaphthoquinone (DNQ) groups (D3-PCL-DNQ). DNQ and sugar groups were modularly synthesized by utilizing click chemistry. As a result, the DOX anticancer drug could be released in a controlled manner by changing the light irradiation time because of the light-triggered disruption of micelles in an aqueous solution. The sugar-coated micelles further demonstrated specific binding with lectins, rendering them useful for targeted drug delivery vesicles [186]. A combination of sugar-based targeting and two-photon sensitivity within the same nanocarrier was described. In such constructs, the light-responsive groups could be modularly conjugated and/or altered. Kabanov and coworkers described a very attractive concept for designing photosensitive polymeric nanocarriers through the electrostatic association between an azobenzene-containing surfactant and the PEG-b-poly(acrylic acid) double-hydrophilic block ionomer [187]. Such complexes self-assembled in aqueous solution and formed vesicle-like aggregates composed of a PEG corona and a poly (acrylic acid) shell attached to azobenzene. The photoisomerization of azobenzene, triggered by light, reversibly altered the amphiphilicity of the surfactants and induced the vesicle disassembly.

Similarly, two photo-responsive core/shell NPs based on hyperbranched polyglycerol (hPG) were synthesized to control DNA release. The shell was composed either of bis-(3-aminopropyl) methylamine (AMPA) or pentaethylenehexamine (PEHA) derivatives and was attached to the hPG core with a photo-responsive o-nitrobenzyl linker. The cleavage was complete by irradiation at 350 nm for 2 min, which showed the high potential of light-sensitive, multivalent amine-functionalized polymers for drug and gene-delivery applications [187].

## 8. Photosensitive Nanofibers

Polymeric nanofibers have rapidly become widely utilized nanostructured materials with high structural integrity. Because of their large specific surface, low weight, chemical specificity, and mechanical flexibility have already been used in numerous areas. Polymeric nanofibers have used biomolecules conjugated with photostimulation to improve their specificity. Several studies demonstrate photocage molecules in nanofibers [188,189,190], but very few are functionalized with biomolecules. In 2012, Ogawa et al. developed a novel sugar-decorated nanofiber prepared by self-assembly of low molecular weight hydrogelators composed of azobenzene and disaccharide lactones. Lectin-binding and cell adhesion assays revealed that the nonreducing ends of the conjugated sugar moieties were exposed on the surfaces of self-assembled nanofibrous hydrogels, effectively recognized by the corresponding lectins. In addition, photoisomerization of azobenzene under UV-irradiation induced the sol-gel hydrogel transition [191].

Henke et al. provided a facile and scalable strategy for preparing nanostructured biotinylated materials with porphyrin photosensitizers by a three-step functionalization of pristine electrospun polystyrene nanofiber membranes. It opened up numerous possibilities for functionalizing surfaces of polystyrene nanofiber membranes with biologically active compounds, as biotin residues strongly bind streptavidin and avidin derivatives. A streptavidin-HRP conjugate, used as a model compound, demonstrated to preserve enzymatic activity after binding to the nanofiber membranes enriched by an ionically attached cationic photosensitizer, generating antimicrobial singlet oxygen under the exclusive control of visible light [192].

## 9. Current Challenges, Opportunities, and Concluding Remarks

This review thoroughly summarized the development of light-induced functional nanocarriers, highlighting their potential as a promising therapeutic strategy for site-specific delivery of drugs. Significantly challenges still exist when designing functional polymeric nanocarriers to exhibit biocompatibility and biodegradability with innocuous degradation products. Reproducible features via synthesis, functionalization, or crosslinking are mandatory.

The creative design of photocontrolled NPs has allowed control over the drug delivery process using noninvasive and spatial stimulation. Yet, its potential for translational applications and convenience is still an open question to be addressed, e.g., UV light has substantial limitations for in vitro and in vivo applications. Many biological molecules absorb these energetic wavelengths directly, preventing substantial tissue penetration and causing undesirable photochemical reactions. In addition, a challenge to achieve the controllable and reproducible fabrication of the light-activatable polymeric nanoformulations is the use of organic solvents, exotic catalysts, free radicals, and transition metal complexes that must be minimized or eliminated during material synthesis.

UV-vis and visible light to stimulate unspecific LRNs are predominant in literature. However, the use of deeper tissue penetrating NIR-light or enhancement of NPs specificity by using coating biomolecules promises to advance this technology towards the clinic. Complex macromolecular architectures and efficient coupling methods conjugated to the discovery of biologically active ligands may offer opportunities to get a vast collection of targeted systems. Their successful application against many different pathologies has demonstrated their great potential for developing personalized nanomedicines of the future.

Implementation of functionalized nanocarriers in patients requires the previous investigation of safety, biodistribution, and pharmacokinetics/pharmacodynamics in multiple animal species. However, the cost could represent a limitation for translation from the bench to the clinical. By handling nanocarriers’ surface characteristics and stealth properties, they can be transformed into smart platforms containing therapeutic and imaging agents for delivering drugs to specific cells and tissues and providing alternatives of controlled-release therapy. The continuous ongoing research on functionalized NPs envisions to improve the prevention, diagnosis and treatment of diseases. Photocontrolled functionalized systems allow unprecedented control over the delivery process using a noninvasive and spatially and temporally controllable external stimulus, holding the potential for site-specific drug delivery versatile alternatives.

## Figures and Tables

**Table 1:** Functionalization of photosensitive nanocarriers.

Method	Advantages	Disadvantages	Interactions/Example	References
Physical adsorption	The most simple. Less aggressive. Reagent-free.	Less stable. Less reproducible.	Van der Waals. Hydrogen bonding. Hydrophobic interactions. Electrostatic interactions.	[56,77]
Entrapment	3D-conformational structure of ligands and drug biomolecules remain almost unaltered, keeping the biological activity.	Poor retention. Diffusional barrier. Limited molecules transport & longer response time.	Gel (polyacrylamide) Silicone, Jelly Chitosan hydrogel	[80]
Cross-linking	Simple Additional stability by intermolecular linking.	Alter the 3D conformation of biomolecules and active center of enzymes. Induce restriction in molecules diffusion.	Glutaraldehyde, Hexamethylene diisocyanate,	[56,77,83]
Biocomposite	Improved properties.	Stability of bioreceptors in solvents and matrix	Embedded active principle in a complex matrix	[56]
Covalent linking	Better stability (pH, ionic strength and temperature). Longer lifetime.	Longer protocols. Lower bioactivity.	Through functional groups (NH_2_, COOH, OH, SH, Ph-OH).	[86,93]

**Table 2:** Summary of surface chemistries to functionalize NPs.

Functional Group	Reactive Group	Reaction	Conditions	Ref.
**Carboxylic** **(R-COOH)**	Carbodiimide 1. EDC (up) 2. EDC/NHS (down)	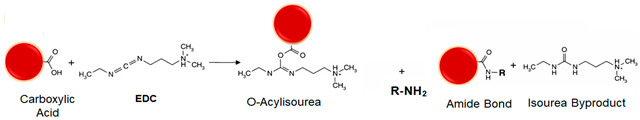 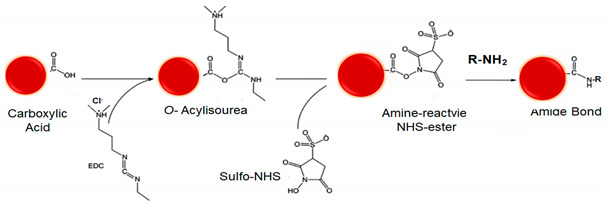	1. Most efficient in pH 4.5 acidic conditions. Carboxyl- and amine-containing buffers must be avoided. 4-morpholinoethanesulfonic acid (MES) buffer is a suitable reaction buffer. Phosphate buffers at neutral pH (up to 7.2) conditions are compatible with the reaction chemistry but with lower efficiency. Increasing the amount of EDC can compensate for the reduced efficiency. 2. It allows for efficient conjugation to primary amines at physiologic pH.	[56,100]
	3. Cyclic carbodiimide (DCC)	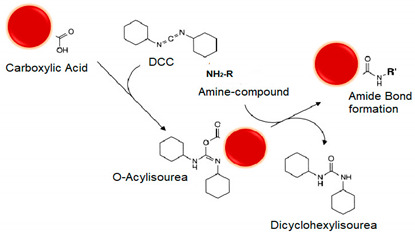	Water-free.	[97,98,100]
**Carbonyls ** **(R-CHO,** **R-CO-R’)**	4. Aldehyde and cetone	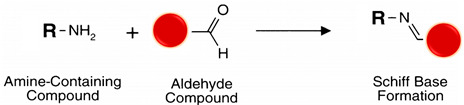	Aldehydes and ketones can react with primary and secondary amines to form Schiff bases. A dehydration reaction yields an imine. The formation of Schiff bases is enhanced at alkaline pH values, but they are still not stable enough to use for crosslinking applications.	[101]
	5. Hydrazide	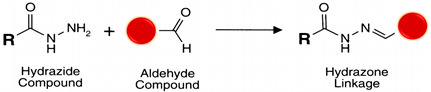	Aldehydes created by periodate-oxidation of sugars in biological samples react with hydrazides at pH 5–7 to form hydrazone bonds.	[102,103]
	6. Alkoxyamine	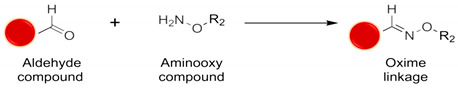	pH 6.5–7.5	[56,104]
	7. Reductive amination	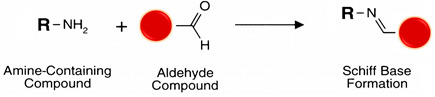 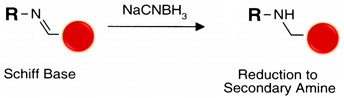	Reduction with sodium cyanoborohydride is necessary to stabilize the Shiff base initially formed.	[77]
**Amine** **(-NH_2_)**	8. Succinimide	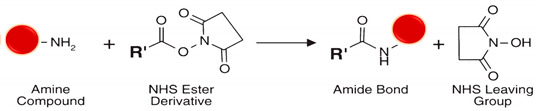	Most commonly performed in phosphate, carbonate-bicarbonate, HEPES or borate buffers at pH 7.2–8.5 for 30 min to 4 h at r.t. or 4 °C. Primary amine buffers such as Tris (TBS) may compete for the reaction, but, in some cases, they are useful to stop it.	[105,107,109]
	9. Imidoester	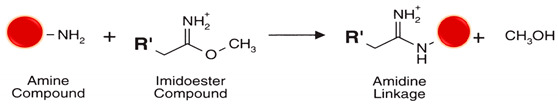	Best done in amine-free, alkaline conditions (pH 10), e.g., borate buffer. Reaction conditions below pH 10 may result in side reactions.	[56,109]
**Sulfhydryl** **(-SH)**	10. Maleimide	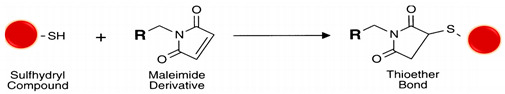	At near neutral conditions (pH 6.5–7.5). At higher pH > 8.5 the reaction favors primary amines and speed-up the maleimide hydrolysis rate to a non-reactive maleamic acid. Tyrosines, histidines or methionines do not react with maleimides. Thiols from most reducing agents must be avoided since they compete for coupling sites. Yet, as TCEP does not contain thiols, it does not have been removed from these reactions.	[108,110,112]
	11. Haloacetyl	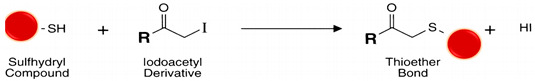	Physiologic to alkaline conditions (pH 7.2–9) in the dark. Imidazoles interact with iodoacetyl groups at pH 6.9–7.0, but it takes more than a week. Histidyl side chains and amino groups react in the unprotonated form with iodoacetyl groups above pH 5 and pH 7, respectively	[56,83]
	12. Pyrioyldisulfide	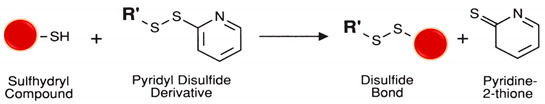	A broad pH range, pH 4–5 being the optimum. The disulfide exchange can be at physiologic pH but with a reaction rate slower than in acidic conditions. Releasing 2-pyridyldithiol that can be monitored by spectrophotometry at 343 nm.	[114]
**Photo-reactive**	13. Aryl azide	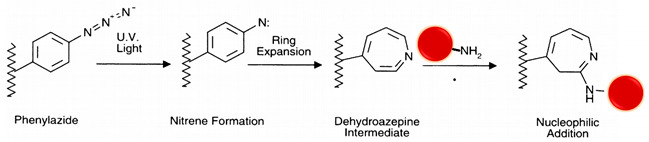	Possible in a variety of amine-free buffer conditions. Avoid exposition to light and reducing agents (they prevent photo-activation). Short-wavelength UV light to activate simple phenyl azides and long UV light for nitrophenyl azides.	[54,117]
	14. Diazirine	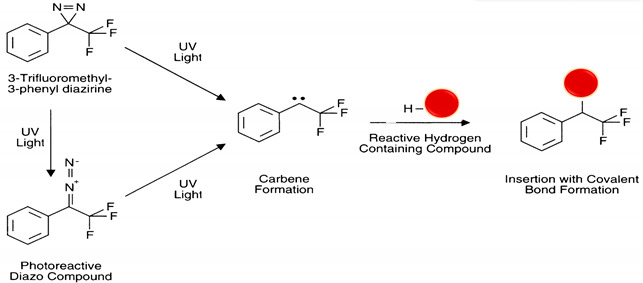	Activated with long-wave UV light (330–370 nm).	[123,124]
**Azide** **(-N_3_)**	15. Phosphine	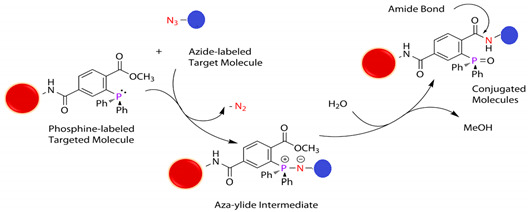	Phosphine-activated molecules react with azide-labeled target molecules to form aza-ylide intermediates, but they rapidly rearrange in aqueous conditions to form stable amide bonds.	[117,118,132]
**Hydroxyl** **(-OH)**	16. Isocyanate	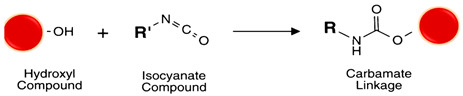	Nonaqueous.	[56,123,124]

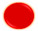
 It schematizes the LRNs and 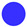 azide-labeled target.

**Table 3:** General characterization techniques of LRNs.

Parameter	Method	Comment	Ref
Particle size	Dynamic light scattering (DLS)	Based on the scattering of light caused by the Brownian movement of the particles.	[139]
	Scanning electron microscopy (SEM)	Observe the morphological state by direct visualization.	[140,141,142]
	Transmission electron microscopy (TEM)		
	Atomic force microscopy (AFM)		
Shape	Microscopy techniques (SEM, TEM, AFM)	Generate images of individual NPs to characterize their shape, size, and location.	[141,143]
	Energy-dispersive X-Ray spectroscopy (EDS)	An electron beam hits the sample, exciting an electron in an inner shell, causing its ejection and forming an electron-hole in the electronic structure of the element.	[140,144]
	X-ray photoelectron spectroscopy (XPS)	Irradiate a material with a beam of X-rays such as a typical Al Kα or Mg Kα source while simultaneously measuring the kinetic energy and number of electrons that escape from atoms on the surface of the material being analyzed.	[145]
	X-ray diffraction analysis (XRD)	It is produced by constructive interference of a monochromatic beam of X-rays scattered at specific angles from each set of lattice planes in a sample. The peak intensities are determined by the atomic positions within the lattice planes.	[141,146,147]
Surface charge	Electrophoretic light scattering (ELS)	It is measured through the mobility of the charged particles under an electric field that indirectly correlates with the surface charge.	[147]
Surface properties	AFM, contact angle measurement (CAM)	CAM has long been used as a criterion of static hydrophobicity of solid surfaces. It is a simple-to-adopt method for surface hydrophobicity analysis based on the sessile drop Young–Laplace method. AFM has been shown to reveal surface energies and hydrophilic or hydrophobic characteristics of the interacting surfaces.	[148]
	Nuclear magnetic resonance spectroscopy (NMR)	It can be defined as an indispensable tool that applies a magnetic field to an atomic nucleus (e.g., the most common stable isotopes 1H, 13C, 15N) and radiofrequency pulses to characterize the resonant frequency of that atomic nucleus according to its chemical or environmental surroundings.	[149]
Thermodynamic properties	Differential scanning calorimetry (DSC) Thermogravimetry (TG)	Give information about the crystallographic structure, chemical composition, and physical properties.	[150]
Encapsulation efficiency, cargo release	Ultraviolet-visible spectrophotometry (UV-Vis), high-performance liquid chromatography (HPLC) Ultracentrifugation, ultrafiltration, gel filtration	Use classical quantification methodologies according to the characteristics of the drug to be encapsulated.	[151]

## Data Availability

Not applicable.

## References

[1] Zhao W., Zhao Y., Wang Q., Liu T., Sun J., Zhang R. (2019). Remote Light-Responsive Nanocarriers for Controlled Drug Delivery: Advances and Perspectives. Small.

[2] Hou W., Liu R., Bi S., He Q., Wang H., Gu J. (2020). Photo-Responsive Polymersomes as Drug Delivery. Molecules.

[3] Gai S., Yang G., Yang P., He F., Lin J., Jin D., Xing B. (2018). Recent Advances in Functional Nanomaterials for Light–Triggered Cancer Therapy. Nano Today.

[4] Avvakumova S., Colombo M., Tortora P., Prosperi D. (2014). Biotechnological Approaches toward Nanoparticle Biofunctionalization. Trends Biotechnol..

[5] Zhou Y., Chen R., Yang H., Bao C., Fan J., Wang C., Lin Q., Zhu L. (2020). Light-Responsive Polymersomes with a Charge-Switch for Targeted Drug Delivery. J. Mater. Chem. B..

[6] Zhang F., Santos H.A. (2019). Photosensitive Materials for Constructing On-Demanded Drug-Release Systems. Photoactive Inorg. Nanoparticles Surf. Compos. Nanosyst. Funct..

[7] Mi P. (2020). Stimuli-Responsive Nanocarriers for Drug Delivery, Tumor Imaging, Therapy and Theranostics. Theranostics.

[8] Zielinska A., Carreiró F., Oliveira A.M., Neves A., Pires B., Nagasamy Venkatesh D., Durazzo A., Lucarini M., Eder P., Silva A.M. (2020). Polymeric Nanoparticles: Production, Characterization, Toxicology and Ecotoxicology. Molecules.

[9] Soppimath K.S., Aminabhavi T.M., Kulkarni A.R., Rudzinski W.E. (2001). Biodegradable Polymeric Nanoparticles as Drug Delivery Devices. J. Control. Release.

[10] Hickey J., Santos J., Williford J., Mao H. (2015). Control of Polymeric Nanoparticle Size to Improve Therapeutic Delivery. J. Control. Release.

[11] Schaffazick S.R., Pohlmann A.R., Dalla-Costa T., Guterres S.S. (2003). Freeze-Drying Polymeric Colloidal Suspensions: Nanocapsules, Nanospheres and Nanodispersion. A Comparative Study. Eur. J. Pharm. Biopharm..

[12] Crucho C.I., Barros M.T. (2017). Polymeric Nanoparticles: A Study on the Preparation Variables and Characterization Methods. Mater. Sci. Eng. C Mater. Biol. Appl..

[13] Barhoumi A., Liu Q., Kohane D.S. (2015). Ultraviolet Light-Mediated Drug Delivery: Principles, Applications, and Challenges. J. Control. Release.

[14] Marturano V., Cerruti P., Giamberini M., Tylkowski B., Ambrogi V. (2016). Polymers Light-Responsive Polymer Micro-and Nano-Capsules. Polymers.

[15] Ferreira Soares D.C., Domingues S.C., Viana D.B., Tebaldi M.L. (2020). Polymer-Hybrid Nanoparticles: Current Advances in Biomedical Applications. Biomed. Pharmacother..

[16] Nicolas J., Mura S., Brambilla D., Mackiewicz N., Couvreur P. (2013). Design, Functionalization Strategies and Biomedical Applications of Targeted Biodegradable/Biocompatible Polymer-Based Nanocarriers for Drug Delivery. Chem. Soc. Rev..

[17] Subbiah R., Veerapandian M., Yun K.S. (2011). Nanoparticles: Functionalization and Multifunctional Applications in Biomedical Sciences. Curr. Med. Chem..

[18] Cheng Y., Meyers J.D., Broome A.-M., Kenney M.E., Basilion J.P., Burda C. (2011). Deep Penetration of a PDT Drug into Tumors by Noncovalent Drug-Gold Nanoparticle Conjugates. J. Am. Chem. Soc..

[19] Nell K., Fontenot S., Carter T., Warner M.G., Warner C.L., Addleman R.S., Johnson D.W. (2016). Non-Covalent Functionalization of High-Surface Area Nanomaterials: A New Class of Sorbent Materials. Environ. Sci. Nano.

[20] Mahon E., Salvati A., Bombelli F.B., Lynch I., Dawson K.A. (2012). Designing the nanoparticle-biomolecule interface for "targeting and therapeutic delivery". J Control Release.

[21] Nobs L., Buchegger F., Gurny R., Allémann E. (2004). Current Methods for Attaching Targeting Ligands to Liposomes and Nanoparticles. J. Pharm. Sci..

[22] Katz J.S., Burdick J.A. (2010). Light-Responsive Biomaterials: Development and Applications. Macromol. Biosci..

[23] Bansal A., Zhang Y. (2014). Photocontrolled Nanoparticle Delivery Systems for Biomedical Applications. Acc. Chem. Res..

[24] Klán P., Šolomek T., Bochet C.G., Blanc A., Givens R., Rubina M., Popik V., Kostikov A., Wirz J. (2013). Photoremovable Protecting Groups in Chemistry and Biology: Reaction Mechanisms and Efficacy. Chem. Rev..

[25] Weinstain R., Slanina T., Kand D., Klán P. (2020). Visible-to-NIR-Light Activated Release: From Small Molecules to Nanomaterials. Chem. Rev..

[26] Zhao H., Sterner E.S., Coughlin B., Theato P. (2012). O-Nitrobenzyl Alcohol Derivatives: Opportunities in Polymer and Materials Science. ACS Publ..

[27] Yan Q., Han D., Zhao Y. (2013). Main-Chain Photoresponsive Polymers with Controlled Location of Light-Cleavable Units: From Synthetic Strategies to Structural Engineering. Polym. Chem..

[28] Goulet-Hanssens A., Barrett C.J. (2013). Photo-Control of Biological Systems with Azobenzene Polymers. J. Polym. Sci. Part. A Polym. Chem..

[29] Beharry A.A., Woolley G.A. (2011). Azobenzene Photoswitches for Biomolecules. Chem. Soc. Rev..

[30] Andreopoulos F.M., Deible C.R., Stauffer M.T., Weber S.G., Wagner W.R., Beckman E.J., Russell A.J. (1996). Photoscissable Hydrogel Synthesis via Rapid Photopolymerization of Novel PEG-Based Polymers in the Absence of Photoinitiators. J. Am. Chem. Soc..

[31] Zheng Y., Andreopoulos F.M., Micic M., Huo Q., Pham S.M., Leblanc R.M. (2011). A Novel Photoscissile Poly(Ethylene Glycol)-Based Hydrogel. Adv. Funct. Mater..

[32] Zheng Y., Micic M., Mello S.V., Mabrouki M., Andreopoulos F.M., Konka V., Pham S.M., Leblanc R.M. (2002). PEG-Based Hydrogel Synthesis via the Photodimerization of Anthracene Groups. ACS Publ..

[33] Liu G., Liu W., Dong C.M. (2013). UV- and NIR-Responsive Polymeric Nanomedicines for on-Demand Drug Delivery. Polym. Chem..

[34] Yue X., Zhang Q., Dai Z. (2017). Near-Infrared Light-Activatable Polymeric Nanoformulations for Combined Therapy and Imaging of Cancer. Adv. Drug Deliv. Rev..

[35] Alonso-Cristobal P., Oton-Fernandez O., Mendez-Gonzalez D., Fernando Díaz J., Lopez-Cabarcos E., Barasoain I., Rubio-Retama J. (2015). Synthesis, Characterization, and Application in HeLa Cells of an NIR Light Responsive Doxorubicin Delivery System Based on NaYF 4:Yb,Tm@SiO 2-PEG Nanoparticles. ACS Publ..

[36] Ercole F., Davis T.P., Evans R.A. (2010). Photo-Responsive Systems and Biomaterials: Photochromic Polymers, Light-Triggered Self-Assembly, Surface Modification, Fluorescence Modulation and Beyond. Polym. Chem..

[37] Sur S., Rathore A., Dave V., Reddy K.R., Chouhan R.S., Sadhu V. (2019). Recent Developments in Functionalized Polymer Nanoparticles for Efficient Drug Delivery System. Nano Struct. Nano Objects.

[38] Liu X., Chu P., Ding C. (2010). Surface Nano-Functionalization of Biomaterials. Mater. Sci. Eng. R. Rep..

[39] Mout R., Moyano D.F., Rana S., Rotello V.M. (2012). Surface Functionalization of Nanoparticles for Nanomedicine. Chem. Soc. Rev..

[40] Martínez E., Lagunas A., Mills C.A., Rodríguez-Seguí S., Estévez M., Oberhansl S., Comelles J., Samitier J. (2009). Stem Cell Differentiation by Functionalized Micro- and Nanostructured Surfaces. Nanomedicine.

[41] Mirkin C.A., Letsinger R.L., Mucic R.C., Storhoff J.J. (1996). A DNA-Based Method for Rationally Assembling Nanoparticles into Macroscopic Materials. Nature.

[42] Singh U., Morya V., Rajwar A., Chandrasekaran A.R., Datta B., Ghoroi C., Bhatia D. (2020). DNA-Functionalized Nanoparticles for Targeted Biosensing and Biological Applications. ACS Omega.

[43] Liu J., Cao Z., Lu Y. (2009). Functional Nucleic Acid Sensors. Chem. Rev..

[44] Silva A.L., Moura L.I.F., Carreira B., Conniot J., Matos A.I., Peres C., Sainz V., Silva L.C., Gaspar R.S., Florindo H.F. (2018). Functional Moieties for Intracellular Traffic of Nanomaterials. Biomedical Applications of Functionalized Nanomaterials: Concepts, Development and Clinical Translation.

[45] Geerts N., Eiser E. (2010). DNA-Functionalized Colloids: Physical Properties and Applications. Soft Matter.

[46] Guo P. (2011). RNA Nanotechnology: Methods for Synthesis, Conjugation, Assembly and Application of RNA Nanoparticles. Methods.

[47] Itani R., Al Faraj A. (2019). Molecular Sciences SiRNA Conjugated Nanoparticles-A Next Generation Strategy to Treat Lung Cancer. Polymers.

[48] Watanabe K., Ohtsuki T. (2016). Photocontrolled Intracellular RNA Delivery Using Nanoparticles or Carrier–Photosensitizer Conjugates. Prog. Mol. Biol. Transl. Sci..

[49] Zhou L.-Y., Qin Z., Zhu Y.-H., He Z.-Y., Xu T. (2019). Current RNA-Based Therapeutics in Clinical Trials. Curr. Gene Ther..

[50] Nimjee S.M., White R.R., Becker R.C., Sullenger B.A. (2017). Aptamers as Therapeutics. Annu. Rev. Pharmacol. Toxicol..

[51] Oh J.H., Park D.H., Joo J.H., Lee J.S. (2015). Recent advances in chemical functionalization of nanoparticles with biomolecules for analytical applications. Anal Bioanal Chem.

[52] Wu S.-C., Ng K.K.-S., Wong S.-L. (2009). Engineering Monomeric Streptavidin and Its Ligands with Infinite Affinity in Binding but Reversibility in Interaction. Proteins.

[53] Yetisgin A.A., Cetinel S., Zuvin M., Kosar A., Kutlu O. (2020). Therapeutic Nanoparticles and Their Targeted Delivery Applications. Molecules.

[54] Dieterich D.C., Lee J.J., Link A.J., Graumann J., Tirrell D.A., Schuman E.M. (2007). Labeling, Detection and Identification of Newly Synthesized Proteomes with Bioorthogonal Non-Canonical Amino-Acid Tagging. Nat. Protoc..

[55] Shamay Y., Adar L., Ashkenasy G., David A. (2011). Light Induced Drug Delivery into Cancer Cells. Biomaterials.

[56] Hermanson G.T., Hermanson G.T. (2013). Chapter 3—The Reactions of Bioconjugation. Bioconjugate Techniques.

[57] Sun T., Zhang Y.S., Pang B., Hyun D.C., Yang M., Xia Y. (2014). Engineered Nanoparticles for Drug Delivery in Cancer Therapy. Angew. Chemie Int. Ed..

[58] Leiro V., Parreira P., Freitas S.C., Martins M.C.L., Pêgo A.P. (2018). Conjugation Chemistry Principles and Surface Functionalization of Nanomaterials.

[59] Dcona M.M., Sheldon J.E., Mitra D., Hartman M.C.T. (2017). Light Induced Drug Release from a Folic Acid-Drug Conjugate. Bioorganic Med. Chem. Lett..

[60] Liu S., Lämmerhofer M. (2019). Functionalized Gold Nanoparticles for Sample Preparation: A Review. Electrophoresis.

[61] Chen C., Ke J., Edward Zhou X., Yi W., Brunzelle J.S., Li J., Yong E.L., Xu H.E., Melcher K. (2013). Structural Basis for Molecular Recognition of Folic Acid by Folate Receptors. Nature.

[62] Zhang X., Wang F., Sheng J.-L., Sun M.-X. (2018). Advances and Application of DNA-Functionalized Nanoparticles. Curr. Med. Chem..

[63] Dai L., Liu Y., Wang Z., Guo F., Shi D., Zhang B. (2014). One-Pot Facile Synthesis of PEGylated Superparamagnetic Iron Oxide Nanoparticles for MRI Contrast Enhancement. Mater. Sci. Eng. C.

[64] Åkerman M.E., Chan W.C.W., Laakkonen P., Bhatia S.N., Ruoslahti E. (2002). Nanocrystal Targeting in Vivo. Proc. Natl. Acad. Sci. USA.

[65] Maldiney T., Richard C., Seguin J., Wattier N., Bessodes M., Scherman D. (2011). Effect of Core Diameter, Surface Coating, and PEG Chain Length on the Biodistribution of Persistent Luminescence Nanoparticles in Mice. ACS Nano.

[66] Bhadra D., Bhadra S., Jain P., Jain N.K. (2002). Pegnology: A Review of PEG-Ylated Systems. Pharmazie.

[67] Kommareddy S., Tiwari S.B., Amiji M.M. (2005). Long-Circulating Polymeric Nanovectors for Tumor-Selective Gene Delivery. Technol. Cancer Res. Treat..

[68] Patel P., Hanini A., Shah A., Patel D., Patel S., Bhatt P., Pathak Y.V. (2019). Surface Modification of Nanoparticles for Targeted Drug Delivery.

[69] Sánchez A., Mejía S.P., Orozco J. (2020). Recent Advances in Polymeric Nanoparticle-Encapsulated Drugs against Intracellular Infections. Molecules.

[70] Zanganeh S., Spitler R., Erfanzadeh M., Alkilany A.M., Mahmoudi M. (2016). Protein Corona: Opportunities and Challenges. Int. J. Biochem. Cell Biol..

[71] Ritz S., Schö S., Kotman N., Baier G., Musyanovych A., Rg Kuharev J., Landfester K., Rg Schild H., Jahn O., Tenzer S. (2015). Protein Corona of Nanoparticles: Distinct Proteins Regulate the Cellular Uptake. ACS Publ..

[72] Wang J., Jensen U.B., Jensen G.V., Shipovskov S., Balakrishnan V.S., Otzen D., Pedersen J.S., Besenbacher F., Sutherland D.S. (2011). Soft Interactions at Nanoparticles Alter Protein Function and Conformation in a Size Dependent Manner. Nano Lett..

[73] Karakoti A.S., Das S., Thevuthasan S., Seal S. (2011). PEGylated Inorganic Nanoparticles. Angew. Chemie Int. Ed..

[74] Li J., Sun C., Tao W., Cao Z., Qian H., Yang X., Wang J. (2018). Photoinduced PEG Deshielding from ROS-Sensitive Linkage-Bridged Block Copolymer-Based Nanocarriers for on-Demand Drug Delivery. Biomaterials.

[75] Gangopadhyay M., Singh T., Behara K.K., Karwa S., Ghosh S.K., Pradeep Singh N.D. (2015). Coumarin-Containing-Star-Shaped 4-Arm-Polyethylene Glycol: Targeted Fluorescent Organic Nanoparticles for Dual Treatment of Photodynamic Therapy and Chemotherapy. Photochem. Photobiol. Sci..

[76] Tonigold M., Simon J., Estupiñán D., Kokkinopoulou M., Reinholz J., Kintzel U., Kaltbeitzel A., Renz P., Domogalla M.P., Steinbrink K. (2018). Pre-Adsorption of Antibodies Enables Targeting of Nanocarriers despite a Biomolecular Corona. Nat. Nanotechnol..

[77] Conde J., Dias J.T., Grazú V., Moros M., Baptista P.V., de la Fuente J.M. (2014). Revisiting 30 Years of Biofunctionalization and Surface Chemistry of Inorganic Nanoparticles for Nanomedicine. Front. Chem..

[78] Mejía De Los Ríos S.P., Sánchez Toro A., Vásquez V., Orozco Holguín J. (2021). Functional Nanocarriers for Delivering Itraconazole against Fungal Intracellular Infections. Front. Pharmacol..

[79] Parracino M.A., Martín B., Grazú V. (2019). State-of-the-Art Strategies for the Biofunctionalization of Photoactive Inorganic Nanoparticles for Nanomedicine. Photoactive Inorganic Nanoparticles: Surface Composition and Nanosystem Functionality.

[80] Strong L.E., West J.L. (2015). Hydrogel-Coated Near Infrared Absorbing Nanoshells as Light-Responsive Drug Delivery Vehicles. ACS Biomater. Sci. Eng..

[81] Luo Y., Shoichet M.S. (2004). A Photolabile Hydrogel for Guided Three-Dimensional Cell Growth and Migration. Nat. Mater..

[82] Pourjavadi A., Bagherifard M., Doroudian M. (2020). Synthesis of Micelles Based on Chitosan Functionalized with Gold Nanorods as a Light Sensitive Drug Delivery Vehicle. Int. J. Biol. Macromol..

[83] Brinkley M. (1992). A Brief Survey of Methods for Preparing Protein Conjugates with Dyes, Haptens and Crosslinking Reagents. Bioconjug. Chem..

[84] Yakovlev A.A. (2009). Crosslinkers and Their Utilization for Studies of Intermolecular Interactions. Neurochem. J..

[85] Yang L., Tang X., Weisbrod C.R., Munske G.R., Eng J.K., Von Haller P.D., Kaiser N.K., Bruce J.E. (2010). A Photocleavable and Mass Spectrometry Identifiable Cross-Linker for Protein Interaction Studies. Anal. Chem..

[86] Sperling R.A., Parak W.J. (2010). Surface Modification, Functionalization and Bioconjugation of Colloidal Inorganic Nanoparticles. Philos. Trans. R. Soc. A.

[87] Febvay S., Marini D.M., Belcher A.M., Clapham D.E. (2010). Targeted Cytosolic Delivery of Cell-Impermeable Compounds by Nanoparticle-Mediated, Light-Triggered Endosome Disruption. Nano Lett..

[88] Hou J., Liu X., Shen J., Zhao G., Wang P.G. (2012). The impact of click chemistry in medicinal chemistry. Expert Opin Drug Discov..

[89] Delfi M., Ghomi M., Zarrabi A., Mohammadinejad R., Taraghdari Z.B., Ashrafizadeh M., Zare E.N., Agarwal T., Padil V.V.T., Mokhtari B. (2020). Functionalization of Polymers and Nanomaterials for Biomedical Applications: Antimicrobial Platforms and Drug Carriers. Prosthesis.

[90] De La Torre T.Z.G., Herthnek D., Ramachandraiah H., Svedlindh P., Nilsson M., Strømme M. (2011). Evaluation of the Sulfo-Succinimidyl-4-(N-Maleimidomethyl) Cyclohexane-1-Carboxylate Coupling Chemistry for Attachment of Oligonucleotides to Magnetic Nanobeads. J. Nanosci. Nanotechnol..

[91] Liu X., Zhao R., Mao W., Feng H., Liu X., Wong D.K.Y. (2011). Detection of Cortisol at a Gold NanoparticleProtein G-DTBP-Scaffold Modified Electrochemical Immunosensor. Analyst.

[92] Roberts M.J., Bentley M.D., Harris J.M. (2002). Chemistry for Peptide and Protein PEGylation. Adv. Drug Deliv. Rev..

[93] Veronese F.M., Harris J.M. (2002). Introduction and Overview of Peptide and Protein Pegylation. Adv. Drug Deliv. Rev..

[94] Lu X., Zheng C., Xu Y., Su Z. (2005). Disuccinimidyl Suberate Cross-Linked Hemoglobin as a Novel Red Blood Cell Substitute. Sci. China Ser. C Life Sci..

[95] Guo R., Zhang L., Qian H., Li R., Jiang X., Liu B. (2010). Multifunctional Nanocarriers for Cell Imaging, Drug Delivery, and near-IR Photothermal Therapy. Langmuir.

[96] Fischer M.J.E. (2010). Amine Coupling through EDC/NHS: A Practical Approach. Methods Mol. Biol..

[97] Lempens E.H.M., Helms B.A., Merkx M. (2011). Chemoselective Protein and Peptide Immobilization on Biosensor Surfaces. Methods Mol. Biol..

[98] Yamashiro D., Blake J. (1981). Use of Thiol Acids in Peptide Segment Coupling in Non-Aqueous Solvents. Int. J. Pept. Protein Res..

[99] Mena-Giraldo P., Pérez-Buitrago S., Londoño-Berrío M., Ortiz-Trujillo I.C., Hoyos-Palacio L.M., Orozco J. (2020). Photosensitive Nanocarriers for Specific Delivery of Cargo into Cells. Sci. Rep..

[100] Rajabi M., Srinivasan M., Mousa S.A. (2016). Nanobiomaterials in Drug Delivery. Nanobiomaterials in Drug Delivery: Applications of Nanobiomaterials.

[101] Zatsepin T.S., Stetsenko D.A., Gait M.J., Oretskaya T.S. (2005). Use of Carbonyl Group Addition-Elimination Reactions for Synthesis of Nucleic Acid Conjugates. Bioconjug. Chem..

[102] Geoghegan K.F., Stroh J.G. (1992). Site-Directed Conjugation of Nonpeptide Groups to Peptides and Proteins Via Periodate Oxidation of a 2-Amino Alcohol. Application to Modification at N-Terminal Serine. Bioconjug. Chem..

[103] Thumshirn G., Hersel U., Goodman S.L., Kessler H. (2003). Multimeric Cyclic RGD Peptides as Potential Tools for Tumor Targeting: Solid-Phase Peptide Synthesis and Chemoselective Oxime Ligation. Chem. A Eur. J..

[104] Xiong X.B., Mahmud A., Uludaǧ H., Lavasanifar A. (2007). Conjugation of Arginine-Glycine-Aspartic Acid Peptides to Poly(Ethylene Oxide)-b-Poly(ε-Caprolactone) Micelles for Enhanced Intracellular Drug Delivery to Metastatic Tumor Cells. Biomacromolecules.

[105] Tao W., Zeng X., Wu J., Zhu X., Yu X., Zhang X., Zhang J., Liu G., Mei L. (2016). Polydopamine-Based Surface Modification of Novel Nanoparticle-Aptamer Bioconjugates for in Vivo Breast Cancer Targeting and Enhanced Therapeutic Effects. Theranostics.

[106] Staros J.V. (1982). N-Hydroxysulfosuccinimide Active Esters: Bis(N-Hydroxysulfosuccinimide) Esters of Two Dicarboxylic Acids Are Hydrophilic, Membrane-Impermeant, Protein Cross-Linkers. Biochemistry.

[107] Cuatrecasas P., Parikh I. (1972). Adsorbents for Affinity Chromatography. Use of N-Hydroxysuccinimide Esters of Agarose. Biochemistry.

[108] Partis M.D., Griffiths D.G., Roberts G.C., Beechey R.B. (1983). Cross-Linking of Protein by ω-Maleimido Alkanoyl N-Hydroxysuccinimido Esters. J. Protein Chem..

[109] Jijie R., Barras A., Bouckaert J., Dumitrascu N., Szunerits S., Boukherroub R. (2018). Enhanced Antibacterial Activity of Carbon Dots Functionalized with Ampicillin Combined with Visible Light Triggered Photodynamic Effects. Colloids Surf. B Biointerfaces.

[110] Smyth D.G., Blumenfeld O.O., Konigsberg W. (1964). Reactions of N-Ethylmaleimide with Peptides and Amino Acids. Biochem. J..

[111] Brewer C.F., Riehm J.P. (1967). Evidence for Possible Nonspecific Reactions between N-Ethylmaleimide and Proteins. Anal. Biochem..

[112] Maheshwari N., Kumar Atneriya U., Tekade M., Sharma M.C., Elhissi A., Tekade R.K. (2019). Guiding Factors and Surface Modification Strategies for Biomaterials in Pharmaceutical Product Development.

[113] Means G.E., Feeney R.E. (1990). Chemical Modifications of Proteins: History and Applications. Bioconjug. Chem..

[114] King T.P., Li Y., Kochoumian L. (1978). Preparation of Protein Conjugates via Intermolecular Disulfide Bond Formation. Biochemistry.

[115] Han G., Ghosh P., Rotello V.M. (2007). Functionalized Gold Nanoparticles for Drug Delivery. Nanomedicine.

[116] Schuh C., Lomadze N., Rühe J., Kopyshev A., Santer S. (2011). Photomechanical Degrafting of Azo-Functionalized Poly(Methacrylic Acid) (PMAA) Brushes. J. Phys. Chem. B.

[117] Jewett J.C., Bertozzi C.R. (2010). Cu-Free Click Cycloaddition Reactions in Chemical Biology. Chem. Soc. Rev..

[118] Pathak Y. (2019). Surface Modification of Nanoparticles for Targeted Drug Delivery. Surface Modification of Nanoparticles for Targeted Drug Delivery.

[119] Lin J.J., Chen J.S., Huang S.J., Ko J.H., Wang Y.M., Chen T.L., Wang L.F. (2009). Folic Acid-Pluronic F127 Magnetic Nanoparticle Clusters for Combined Targeting, Diagnosis, and Therapy Applications. Biomaterials.

[120] Li Y., Busatto N., Roth P.J. (2021). Perfluorophenyl Azides: Photo, Staudinger, and Multicomponent Postpolymerization Reactions on Homopolymers and PISA-Made Nanoparticles. Macromolecules.

[121] Bethell G.S., Ayers J.S., Hancock W.S., Hearn M.T. (1979). A Novel Method of Activation of Cross-Linked Agaroses with 1,1’-Carbonyldiimidazole Which Gives a Matrix for Affinity Chromatography Devoid of Additional Charged Groups. J. Biol. Chem..

[122] Beauchamp C.O., Gonias S.L., Menapace D.P., Pizzo S.V. (1983). A New Procedure for the Synthesis of Polyethylene Glycol-Protein Adducts; Effects on Function, Receptor Recognition, and Clearance of Superoxide Dismutase, Lactoferrin, and alpha 2-macroglobulin. Anal. Biochem..

[123] Morales-Serna J., Vera A., Paleo E., García-Ríos E. (2010). Using Benzotriazole Esters as a Strategy in the Esterification of Tertiary Alcohols. Synthesis.

[124] Xu Y., Miller M.J. (1998). Total Syntheses of Mycobactin Analogues as Potent Antimycobacterial Agents Using a Minimal Protecting Group Strategy. J. Org. Chem..

[125] Inanaga J., Hirata K., Saeki H., Katsuki T., Yamaguchi M. (1979). A Rapid Esterification by Means of Mixed Anhydride and Its Application to Large-Ring Lactonization. Bull. Chem. Soc. Jpn..

[126] Oshiro-Junior J., Sato M., Boni F., Santos K.L.M. (2020). Phthalocyanine-Loaded Nanostructured Lipid Carriers Functionalized with Folic Acid for Photodynamic Therapy. Mater. Sci. Eng. C Mater. Biol. Appl..

[127] Einafshar E., Asl A.H., Nia A.H., Mohammadi M., Malekzadeh A., Ramezani M. (2018). New Cyclodextrin-Based Nanocarriers for Drug Delivery and Phototherapy Using an Irinotecan Metabolite. Carbohydr. Polym..

[128] Barrera C., Herrera A.P., Bezares N., Fachini E., Olayo-Vales R., Hinestroza J.P., Rinaldi C. (2012). Effect of Poly (Ethylene Oxide)-Silane Graft Molecular Weight on the Colloidal Properties of Iron Oxide Nanoparticles for Biomedical Applications. J. Colloid Interface Sci..

[129] Aslan K., Luhrs C.C., Pérez-Luna V.H. (2004). Controlled and Reversible Aggregation of Biotinylated Gold Nanoparticles with Streptavidin. ACS Publ..

[130] Thanh N.T.K., Green L.A.W. (2010). Functionalisation of Nanoparticles for Biomedical Applications. Nano Today.

[131] Tannous B.A., Grimm J., Perry K.F., Chen J.W., Weissleder R., Breakefield X.O. (2006). Metabolic Biotinylation of Cell Surface Receptors for in Vivo Imaging. Nat. Methods.

[132] Li Q.L., Sun Y., Sun Y.L., Wen J., Zhou Y., Bing Q.M., Isaacs L.D., Jin Y., Gao H., Yang Y.W. (2014). Mesoporous Silica Nanoparticles Coated by Layer-by-Layer Self-Assembly Using Cucurbit[7]Uril for in Vitro and in Vivo Anticancer Drug Release. Chem. Mater..

[133] Richardson J.J., Cui J., Björnmalm M., Braunger J.A., Ejima H., Caruso F. (2016). Innovation in Layer-by-Layer Assembly. Chem. Rev..

[134] Huang G., Zhang K.L., Chen S., Li S.H., Wang L.L., Wang L.P., Liu R., Gao J., Yang H.H. (2017). Manganese-Iron Layered Double Hydroxide: A Theranostic Nanoplatform with PH-Responsive MRI Contrast Enhancement and Drug Release. J. Mater. Chem. B.

[135] Richardson J.J., Björnmalm M., Caruso F. (2015). Multilayer Assembly.Technology-Driven Layer-by-Layer Assembly of Nanofilms. Science.

[136] Carvalho J.A., da Silva Abreu A., Tedesco A.C., Junior M.B., Simioni A.R. (2019). Functionalized Photosensitive Gelatin Nanoparticles for Drug Delivery Application. J. Biomater. Sci. Polym. Ed..

[137] Wei X., Beltrán-Gastélum M., Karshalev E., Esteban-Fernández de Ávila B., Zhou J., Ran D., Angsantikul P., Fang R.H., Wang J., Zhang L. (2019). Biomimetic Micromotor Enables Active Delivery of Antigens for Oral Vaccination. Nano Lett..

[138] Jain A., Jain S.K., Ganesh N., Barve J., Beg A.M. (2010). Design and Development of Ligand-Appended Polysaccharidic Nanoparticles for the Delivery of Oxaliplatin in Colorectal Cancer. Nanomed. Nanotechnol. Biol. Med..

[139] Gaumet M., Vargas A., Gurny R., Delie F. (2008). Nanoparticles for Drug Delivery: The Need for Precision in Reporting Particle Size Parameters. Eur. J. Pharm. Biopharm..

[140] Pathak Y., Thassu D. (2016). Microscopic and Spectroscopi Characterization of Nanoparticles. Press Drug Delivery Nanoparticles Formulation and Characterization.

[141] Bandyopadhyay S., Peralta-Videa J.R., Hernandez-Viezcas J.A., Montes M.O., Keller A.A., Gardea-Torresdey J.L. (2012). Microscopic and Spectroscopic Methods Applied to the Measurements of Nanoparticles in the Environment. Appl. Spectrosc. Rev..

[142] Bootz A., Vogel V., Schubert D., Kreuter J. (2004). Comparison of Scanning Electron Microscopy, Dynamic Light Scattering and Analytical Ultracentrifugation for the Sizing of Poly(Butyl Cyanoacrylate) Nanoparticles. Eur. J. Pharm. Biopharm..

[143] Lin P.C., Lin S., Wang P.C., Sridhar R. (2014). Techniques for Physicochemical Characterization of Nanomaterials. Biotechnol. Adv..

[144] Oikawa T. (2006). Energy Dispersive X-Ray Spectroscopy. Jpn. J. Tribol..

[145] Korin E., Froumin N., Cohen S. (2017). Surface Analysis of Nanocomplexes by X-Ray Photoelectron Spectroscopy (XPS). ACS Biomater. Sci. Eng..

[146] Epp J. (2016). X-Ray Diffraction (XRD) Techniques for Materials Characterization. Materials Characterization Using Nondestructive Evaluation (NDE) Methods.

[147] Grobelny J., DelRio F.W., Pradeep N., Kim D.I., Hackley V.A., Cook R.F. (2011). Size Measurement of Nanoparticles Using Atomic Force Microscopy. Methods Mol. Biol..

[148] Fu W., Zhang W. (2018). Measurement of the Surface Hydrophobicity of Engineered Nanoparticles Using an Atomic Force Microscope. Phys. Chem. Chem. Phys..

[149] Sanders J.A. (1995). Magnetic Resonance Spectroscopy. Functional Brain Imaging.

[150] Calvo P., Vila-Jato J.Ä.L., Alonso M.J. (1996). Comparative in Vitro Evaluation of Several Colloidal Systems, Nanoparticles, Nanocapsules, and Nanoemulsions, as Ocular Drug Carriers. J. Pharm. Sci..

[151] Patravale V., Dandekar P., Jain R. (2012). Characterization Techniques for Nanoparticulate Carriers. Nanoparticulate Drug Delivery.

[152] Kumari A., Yadav S., Biointerfaces S.Y. (2010). Biodegradable Polymeric Nanoparticles Based Drug Delivery Systems. Colloids Surf. B Biointerfaces.

[153] Dvir T., Banghart M.R., Timko B.P., Langer R., Kohane D.S. (2010). Photo-Targeted Nanoparticles. Nano Lett..

[154] Kim H.M., Cho B.R. (2015). Small-Molecule Two-Photon Probes for Bioimaging Applications. Chem. Rev..

[155] Hühn D., Kantner K., Geidel C., Brandholt S., De Cock I., Soenen S.J.H., Riveragil P., Montenegro J.M., Braeckmans K., Müllen K. (2013). Polymer-Coated Nanoparticles Interacting with Proteins and Cells: Focusing on the Sign of the Net Charge. ACS Nano.

[156] Techane S., Baer D.R., Castner D.G. (2011). Simulation and Modeling of Self-Assembled Monolayers of Carboxylic Acid Thiols on Flat and Nanoparticle Gold Surfaces. Anal. Chem.

[157] Walkey C.D., Olsen J.B., Guo H., Emili A., Chan W.C.W. (2012). Nanoparticle Size and Surface Chemistry Determine Serum Protein Adsorption and Macrophage Uptake. J. Am. Chem. Soc..

[158] Sapsford K.E., Tyner K.M., Dair B.J., Deschamps J.R., Medintz I.L. (2011). Analyzing Nanomaterial Bioconjugates: A Review of Current and Emerging Purification and Characterization Techniques. Anal. Chem..

[159] Zhang F., Feng S., Hélder A., Prieto J.P., Bejar M.G. (2019). Photosensitive Materials for Constructing On-Demanded Drug-Release Systems. Photoactive Inorganic Nanoparticles: Surface Composition and Nanosystem Functionality.

[160] Chen S., Bian Q., Wang P., Zheng X., Lv L., Dang Z., Wang G. (2017). Photo, PH and Redox Multi-Responsive Nanogels for Drug Delivery and Fluorescence Cell Imaging. Polym. Chem..

[161] Paus C., Van Der Voort R., Cambi A., Shi Y. (2021). Nanomedicine in Cancer Therapy: Promises and Hurdles of Polymeric Nanoparticles Exploration of Medicine. Explor. Med..

[162] Sanità G., Carrese B., Lamberti A. (2020). Nanoparticle Surface Functionalization: How to Improve Biocompatibility and Cellular Internalization. Front. Mol. Biosci..

[163] Guerrini L., Alvarez-Puebla R.A., Pazos-Perez N. (2018). Materials Surface Modifications of Nanoparticles for Stability in Biological Fluids. Materials.

[164] Asanuma H., Liang X., Liu M., Nishioka H., Matsunaga D., Komiyama M. (2007). Synthesis of Azobenzene-Tethered DNA for Reversible Photo-Regulation of DNA Functions: Hybridization and Transcription. Nat. Protoc..

[165] Nishioka H., Liang X., Asanuma H. (2010). Effect of the Ortho Modification of Azobenzene on the Photoregulatory Efficiency of DNA Hybridization and the Thermal Stability of Its Eis Form. Chem. A Eur. J..

[166] Cheng C.C., Gebeyehu B.T., Huang S.Y., Abebe Alemayehu Y., Sun Y.T., Lai Y.C., Chang Y.H., Lai J.Y., Lee D.J. (2019). Entrapment of an Adenine Derivative by a Photo-Irradiated Uracil-Functionalized Micelle Confers Controlled Self-Assembly Behavior. J. Colloid Interface Sci..

[167] Kang H., Liu H., Zhang X., Yan J., Zhu Z., Peng L., Yang H., Kim Y., Tan W. (2011). Photoresponsive DNA-Cross-Linked Hydrogels for Controllable Release and Cancer Therapy. Langmuir.

[168] Hoffman A.S. (2002). Hydrogels for Biomedical Applications. Adv. Drug Deliv. Rev..

[169] Ru Choi J. (2019). Recent Advances in Photo-Crosslinkable Hydrogels for Biomedical Applications. Futur. Sci..

[170] Wylie R.G., Shoichet M.S. (2008). Two-Photon Micropatterning of Amines within an Agarose Hydrogel. J. Mater. Chem..

[171] Han K., Lei Q., Jia H.Z., Wang S.B., Yin W.N., Chen W.H., Cheng S.X., Zhang X.Z. (2015). A Tumor Targeted Chimeric Peptide for Synergistic Endosomal Escape and Therapy by Dual-Stage Light Manipulation. Wiley Online Libr..

[172] Yang Y., Xie X., Yang Y., Li Z., Yu F., Gong W., Li Y., Zhang H., Wang Z., Mei X. (2016). Polymer Nanoparticles Modified with Photo- and PH-Dual-Responsive Polypeptides for Enhanced and Targeted Cancer Therapy. Mol. Pharm..

[173] Hong G., Zou Y., Antaris A.L., Diao S., Wu D., Cheng K., Zhang X., Chen C., Liu B., He Y. (2014). Ultrafast Fluorescence Imaging in Vivo with Conjugated Polymer Fluorophores in the Second Near-Infrared Window. Nat. Commun..

[174] Xiong J., Feng J.L., Qiu L., Gao Z., Li P., Pang L., Zhang Z. (2019). SDF-1-Loaded PLGA Nanoparticles for the Targeted Photoacoustic Imaging and Photothermal Therapy of Metastatic Lymph Nodes in Tongue Squamous Cell Carcinoma. Int. J. Pharm..

[175] Cai X., Prominski A., Lin Y., Ankenbruck N., Rosenberg J., Chen M., Shi J., Chang E.B., Penaloza-Macmaster P., Tian B. (2020). A Neutralizing Antibody-Conjugated Photothermal Nanoparticle Captures and Inactivates SARS-CoV-2. biorxiv.

[176] Lv Y., Li F., Wang S., Lu G., Bao W., Wang Y., Tian Z., Wei W., Ma G. (2021). Near-Infrared Light–Triggered Platelet Arsenal for Combined Photothermal-Immunotherapy against Cancer. Sci. Adv..

[177] Zhao B., Moore J.S., Beebe D.J. (2001). Surface-Directed Liquid Flow inside Microchannels. Science.

[178] Kaneko S., Yamaguchi K., Nakanishi J. (2013). Dynamic Substrate Based on Photocleavable Poly(Ethylene Glycol): Zeta Potential Determines the Capability of Geometrical Cell Confinement. Langmuir.

[179] Yamaguchi S., Yamahira S., Kikuchi K., Sumaru K., Kanamori T., Nagamune T. (2012). Cell Patterning Photocontrollable Dynamic Micropatterning of Non-Adherent Mammalian Cells Using a Photocleavable Poly(Ethylene Glycol) Lipid. Angew. Chemie.

[180] Park S.J., Park W., Na K. (2013). Photo-Activatable Ternary Complex Based on a Multifunctional Shielding Material for Targeted ShRNA Delivery in Cancer Treatment. Biomaterials.

[181] Ma D., Lin Q.M., Zhang L.M., Liang Y.Y., Xue W. (2014). A Star-Shaped Porphyrin-Arginine Functionalized Poly(l-Lysine) Copolymer for Photo-Enhanced Drug and Gene Co-Delivery. Biomaterials.

[182] Yuan Z., Zhao D., Yi X., Zhuo R. (2014). Steric Protected and Illumination-Activated Tumor Targeting Accessory for Endowing Drug-Delivery Systems with Tumor Selectivity. Adv. Funct. Mater..

[183] Liu F., Chen Y., Li Y., Guo Y., Cao Y., Li P., Wang Z., Gong Y., Ran H. (2018). Folate-Receptor-Targeted Laser-Activable Poly (Lactide-Co-Glycolic Acid) Nanoparticles Loaded with Paclitaxel/Indocyanine Green for Photoacoustic/Ultrasound. Int. J. Nanomed..

[184] Senthilkumar T., Zhou L., Gu Q., Liu L., Lv F., Wang S. (2018). Conjugated Polymer Nanoparticles with Appended Photo-Responsive Units for Controlled Drug Delivery, Release, and Imaging. Angew. Chemie Int. Ed..

[185] Hang C., Zou Y., Zhong Y., Zhong Z., Meng F. (2017). NIR and UV-Responsive Degradable Hyaluronic Acid Nanogels for CD44-Targeted and Remotely Triggered Intracellular Doxorubicin Delivery. Colloids Surfaces B Biointerfaces.

[186] Sun L., Yang Y., Dong C., Wei Y. (2011). Two-photon-sensitive and Sugar-targeted Nanocarriers from Degradable and Dendritic Amphiphiles. Small.

[187] Wang Y., Han P., Xu H., Wang Z., Zhang X., Kabanov A.V. (2010). Photocontrolled Self-Assembly and Disassembly of Block Ionomer Complex Vesicles: A Facile Approach toward Supramolecular Polymer Nanocontainers. Langmuir.

[188] Nezhadghaffar-Borhani E., Abdollahi A., Roghani-Mamaqani H., Salami-Kalajahi M. (2021). Photoswitchable Surface Wettability of Ultrahydrophobic Nanofibrous Coatings Composed of Spiropyran-Acrylic Copolymers. J. Colloid Interface Sci..

[189] Zou L., Han D., Yuan Z., Chang D., Ma X. (2018). A Self-Assembled Photoresponsive Gel Consisting of Chiral Nanofibers. Beilstein J. Org. Chem..

[190] Karimipour K., Keyvan Rad J., Shirvalilou S., Khoei S., Mahdavian A.R. (2021). Spiropyran-Based Photoswitchable Acrylic Nanofibers: A Stimuli-Responsive Substrate for Light Controlled C6 Glioma Cells Attachment/Detachment. Colloids Surfaces B Biointerfaces.

[191] Ogawa Y., Yoshiyama C., Kitaoka T. (2012). Helical Assembly of Azobenzene-Conjugated Carbohydrate Hydrogelators with Specific Affinity for Lectins. Langmuir.

[192] Henke P., Dolanský J., Kubát P., Mosinger J. (2020). Multifunctional Photosensitizing and Biotinylated Polystyrene Nanofiber Membranes/Composites for Binding of Biologically Active Compounds. ACS Appl. Mater. Interfaces.

